# Distinct and shared functions of ALS-associated proteins TDP-43, FUS and TAF15 revealed by multisystem analyses

**DOI:** 10.1038/ncomms12143

**Published:** 2016-07-05

**Authors:** Katannya Kapeli, Gabriel A. Pratt, Anthony Q. Vu, Kasey R. Hutt, Fernando J. Martinez, Balaji Sundararaman, Ranjan Batra, Peter Freese, Nicole J. Lambert, Stephanie C. Huelga, Seung J. Chun, Tiffany Y. Liang, Jeremy Chang, John P. Donohue, Lily Shiue, Jiayu Zhang, Haining Zhu, Franca Cambi, Edward Kasarskis, Shawn Hoon, Manuel Ares Jr., Christopher B. Burge, John Ravits, Frank Rigo, Gene W. Yeo

**Affiliations:** 1 Department of Cellular and Molecular Medicine, University of California at San Diego, La Jolla, California 92093, USA; 2 Stem Cell Program and Institute for Genomic Medicine, University of California at San Diego, La Jolla, California 92093, USA; 3 Department of Physiology, Yong Loo Lin School of Medicine, National University of Singapore, Singapore 117549, Singapore; 4 Department of Bioinformatics and Systems Biology, University of California at San Diego, La Jolla, California 92093, USA; 5 Department of Neurosciences, University of California at San Diego, La Jolla, California 92093, USA; 6 Department of Biology, MIT, Cambridge, Massachusetts 02142, USA; 7 Ionis Pharmaceuticals, Carlsbad, California 92010, USA; 8 Department of Molecular, Cell and Developmental Biology, Sinsheimer Labs, University of California, Santa Cruz, California 95064, USA; 9 Department of Molecular and Cellular Biochemistry, College of Medicine, University of Kentucky, Lexington, Kentucky 40536, USA; 10 Department of Neurology, University of Kentucky, Lexington, Kentucky 40536, USA; 11 Molecular Engineering Laboratory, A*STAR, Singapore 138673, Singapore

## Abstract

The RNA-binding protein (RBP) TAF15 is implicated in amyotrophic lateral sclerosis (ALS). To compare TAF15 function to that of two ALS-associated RBPs, FUS and TDP-43, we integrate CLIP-seq and RNA Bind-N-Seq technologies, and show that TAF15 binds to ∼4,900 RNAs enriched for GGUA motifs in adult mouse brains. TAF15 and FUS exhibit similar binding patterns in introns, are enriched in 3′ untranslated regions and alter genes distinct from TDP-43. However, unlike FUS and TDP-43, TAF15 has a minimal role in alternative splicing. In human neural progenitors, TAF15 and FUS affect turnover of their RNA targets. In human stem cell-derived motor neurons, the RNA profile associated with concomitant loss of both TAF15 and FUS resembles that observed in the presence of the ALS-associated mutation FUS R521G, but contrasts with late-stage sporadic ALS patients. Taken together, our findings reveal convergent and divergent roles for FUS, TAF15 and TDP-43 in RNA metabolism.

## Abstract (web summary)

Abnormal functions of RNA-binding proteins TAF15, FUS and TDP43 are associated with amyotrophic lateral sclerosis. Here, Kapeli *et al*. characterize the RNA targets of TAF15 and identify points of convergence and divergence between the targets of TAF15, FUS and TDP43 in several neuronal systems.

## Graphical abstract

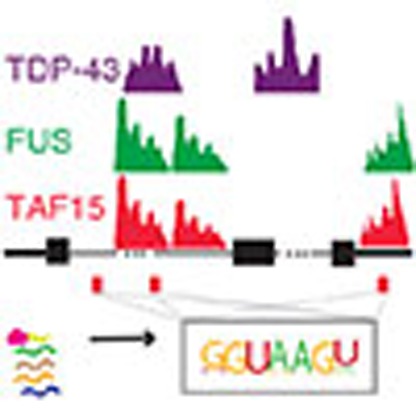

Amyotrophic lateral sclerosis (ALS) is a fatal disease characterized by progressive degeneration of motor neurons (MNs) in the motor cortex, brainstem and spinal cord. Although the precise pathogenesis of ALS remains unknown, aberrant RNA processing appears to be an important contributing factor. The RNA-binding protein (RBP) TAR DNA-binding protein 43 (TDP-43) was initially recognized as a major constituent of pathological ubiquitinated protein aggregates in the brain and spinal cord tissue of patients with sporadic ALS (sALS)^1^,^2^. Dominant mutations in *TDP-43* were subsequently identified in ALS patients^3^,^4^,^5^,^6^,^7^,^8^ with evidence that these mutations were indeed causative of ALS pathogenesis^9^. Shortly thereafter, mutations in the gene encoding another RBP, fused in sarcoma (*FUS*, also known as translocated in liposarcoma or TLS), were identified in a subset of patients with familial ALS and sALS^10^,^11^. Although mutations in *FUS* and *TDP-43* are present in only a small fraction of ALS cases, abnormal activity of FUS and TDP-43 is observed in a large fraction of ALS cases.

The discovery of mutations in the genes encoding *TDP-43* and *FUS* received much attention as these proteins have strikingly similar protein domain architectures^12^. This motivated a search for more structurally similar RBPs as candidate ALS genes and, as a result, mutations in TATA box-binding protein (TBP)-associated factor 15 (*TAF15*) were identified in patients with sALS and familial ALS^13^,^14^. FUS and TAF15 belong to the FET family of heterogeneous nuclear ribonucleoparticle (hnRNP) proteins, which includes Ewing sarcoma breakpoint region 1. As the protein structure of TAF15 is similar to those of FUS and TDP-43 (ref. 13), it was predicted that TAF15 would be functionally similar to these RBPs. Similar to FUS and TDP-43, TAF15 is predominantly localized to the nucleus but shuttles to and from the cytoplasm, participates in transcription, is thought to affect alternative splicing (AS) and has been found to form cytoplasmic inclusions in all FUS-FTLD subtypes and in some sALS patient tissues^13^,^15^,^16^.

Another commonality among TDP-43, FUS and TAF15 is that the vast majority of ALS-associated mutations identified in the genes encoding these RBPs are found in their C-terminal Gly-rich domains. An emerging hypothesis is that mutations within the Gly-rich region of these RBPs promote their pathological aggregation^17^,^18^. Aggregation of FUS, TDP-43 and TAF15 proteins is often accompanied by loss of their nuclear localization; yet it is unclear whether protein aggregation or mislocalization to the cytoplasm is the initiating pathogenic event^19^. In efforts to investigate the normal nuclear function of these RBPs, comprehensive RNA-binding maps of TDP-43, FUS and TAF15 in the normal mouse^20^,^21^,^22^ or human^15^ central nervous system (CNS) have been determined. These studies revealed global roles for TDP-43, FUS and TAF15 in AS and motif specificities for TDP-43 and FUS in the CNS. Furthermore, loss of TDP-43 or FUS expression affects the RNA levels of genes containing long introns^20^,^21^,^22^. Our understanding of FUS, TDP-43 and TAF15 function in RNA processing has primarily come from examining these proteins individually and under different conditions, making comparisons difficult. This approach has limited our understanding of how the activities of these RBPs may converge on common pathways or act in parallel. A systematic comparison of FUS, TDP-43 and TAF15 to determine their shared and unique functions in mature and developing neurons would be valuable in understanding their contribution to development and ultimately disease.

Here we identify 4,873 RNA targets of TAF15 in the mouse brain that reveal a TAF15-binding motif. Expanding on our previous studies^21^,^22^, we find that FUS and TAF15 exhibit similar global RNA interaction profiles *in vivo*, but affect a strikingly small subset of common genes. Unexpectedly, TAF15 influences a small fraction of AS events compared with TDP-43 and FUS in the mouse CNS. In human neural progenitor cells (NPCs), we find that TAF15 and FUS affect the stability of distinct mRNA populations, many of which are bound by TAF15 and FUS. Depletion of TAF15, FUS and TDP-43 in human-induced pluripotent stem cell (iPSC)-derived MNs also affects different genes. Subsets of TAF15 and FUS-regulated mRNAs, including ALS-associated genes, are also differentially expressed in spinal cord MNs dissected from sALS patients and iPSC-derived MNs from ALS patients harbouring a *R521G* mutation in FUS. Taken together, these findings uncover points of functional convergence and divergence of FUS, TAF15 and TDP-43.

## Results

### TAF15 binds RNAs enriched for GGUAAGU motifs *in vivo*

**Figure 1:**
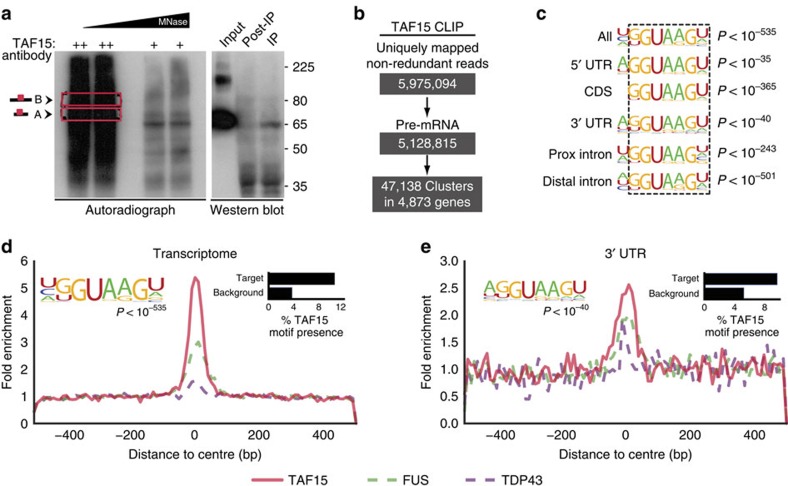
CLIP-seq reveals that TAF15 binds GGUAAGU motifs in the mouse brain. (**a**) Autoradiograph of TAF15 protein–RNA complexes from the mouse brain immunoprecipitated with an antibody against TAF15 (left panel). RNA residing in the regions outlined by the red boxes was recovered for sequencing. TAF15–RNA complexes migrated at the expected size and were efficiently recovered because little protein remained in post-immunoprecipitation lysate (right panel, middle lane). (**b**) Flow chart illustrating CLIP-seq reads analysed to define TAF15 clusters. (**c**) *De novo* sequence motifs enriched above background within the transcriptome or specific genic regions with associated binomial *P* values. (**d**) Positional distribution of the TAF15 motif GGUAAGU within TAF15 (red), FUS (green) or TDP-43 (purple) CLIP clusters. Inset graph shows the per cent enrichment of the TAF15 motif GGUAAGU within TAF15 targets (‘Target') or within the transcriptome (‘Background'). (**e**) Positional distribution analysis of the TAF15 motif GGUAAGU as in **d** but specifically within CLIP clusters residing in 3′ UTRs. Inset graph shows the per cent enrichment of the TAF15 motif GGUAAGU within the 3′ UTRs of TAF15 targets (‘Target') or within the 3′ UTRs of the transcriptome (‘Background').

To identify *in vivo* RNA substrates recognized by TAF15, we performed CLIP (crosslinking immunoprecipitation)-seq in whole-brain tissue from adult mice using a commercially available antibody that specifically recognizes the N terminus of the TAF15 protein. We isolated RNA from low and high molecular weight TAF15 protein–RNA complexes (Fig. 1a, bands A and B, respectively) and converted the RNA into sequencing libraries for transcript identification. No protein–RNA complexes were immunoprecipitated when using nonspecific IgG or in the absence of ultraviolet crosslinking (Supplementary Fig. 1a). Interactions between TAF15 and FUS have previously been detected^23^,^24^. Therefore, we tested whether TAF15 and FUS interact post-cell lysis. Uniquely tagged versions of TAF15 and FUS proteins were expressed separately in HEK293T cells, and upon mixing lysates from these cell lines we found that V5-tagged TAF15 immunoprecipitates Myc-tagged FUS (Supplementary Fig. 1b, lane 10) and *vice versa* (Supplementary Fig. 1b, lane 14). This demonstrated that TAF15 and FUS can physically associate post-cell lysis. For our TAF15 CLIP-seq experiments, the use of ultraviolet-crosslinked cells and highly stringent lysis and wash conditions prevented co-immunoprecipitation of FUS (Supplementary Fig. 1a) and TDP-43 (Supplementary Fig. 1c) with TAF15, ensuring that FUS-RNA and TDP-43-RNA complexes were not inadvertently recovered. Given the high overlap in sequence similarity between the TAF15 target RNAs isolated from the low (band A) and high (band B) molecular weight complexes (Supplementary Fig. 1d), the libraries were combined (Fig. 1b and Supplementary Fig. 1e), resulting in 5.9 million non-redundant sequenced reads that mapped to 13,633 annotated protein-coding pre-mRNAs having more than 10 reads (5,128,815 reads, 85.8%), noncoding genes (139,382 reads, 2.3%) and intergenic regions (706,897 reads, 11.8%) in the mouse genome (mm9).

Using a published cluster-finding algorithm^22^, we identified 47,138 TAF15-binding clusters in 4,873 genes. We applied the HOMER algorithm to these clusters to discover *in vivo* TAF15-binding motifs. The consensus motif GGUAAGU was statistically significantly enriched in TAF15 clusters (Fig. 1c, *P*<10^−535^). Interestingly, this motif is similar to the 5′ splice site sequence, GURAGU (ref. 25); however, enrichment of this motif in both the coding sequence and the 3′ untranslated repeat (UTR) provided evidence that we did not inadvertently extract the 5′ splice site sequence within introns. Distribution analysis also illustrated that the TAF15 motif is enriched within the centre of the CLIP clusters in the transcriptome (Fig. 1d) and also within 3′ UTRs (Fig. 1e). We searched for the TAF15 motif in clustered reads from published FUS (ref. 21) and TDP-43 (ref. 22) CLIP-seq experiments and observed that the TAF15 motif was also enriched in transcriptome-wide FUS CLIP clusters and, to a lesser extent, in TDP-43 CLIP clusters residing in 3′ UTRs (Fig. 1d,e). We conclude that TAF15 interacts with binding sites enriched for a GGUAAGU motif within thousands of genes *in vivo*.

### RNA Bind-n-Seq reveals TAF15 binding to GGUA motif *in vitro*

**Figure 2:**
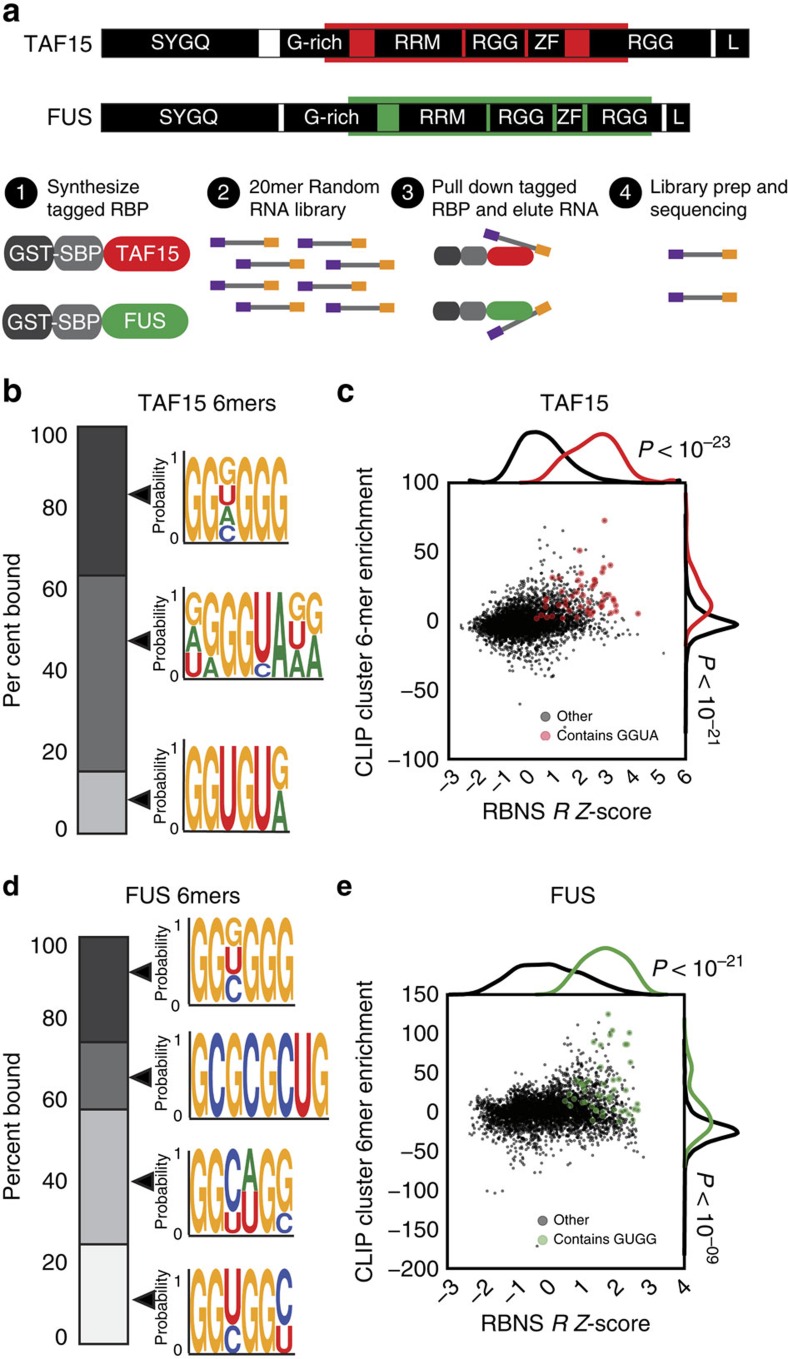
RNA Bind-n-Seq confirms enrichment for GGUA motifs in RNAs that bind TAF15 *in vitro.* (**a**) Experimental overview of RBNS. Truncated versions of TAF15 and FUS (highlighted in red and green, respectively) were tagged and incubated at different concentrations with a diverse pool of RNA oligonucleotides flanked by adapters. The tagged proteins were retrieved with streptavidin-coated beads and bound RNAs were sequenced. Input RNA was sequenced in parallel to quantify the proportions of bound RNA molecules. (**b**) RNA-binding preferences for truncated TAF15 shown as motif logos made from aligning RBNS 6mers weighted by their enrichments. Motif proportions were determined by summing the enrichments of each motif's aligned 6mers. (**c**) Scatter plot correlating the per cent enrichment above background of 6mers in TAF15 mouse brain CLIP-seq versus TAF15 RBNS *R Z*-scores. Red dots represent all significant 6mers containing the GGUA motif. Histograms show normalized distributions of 6mers containing (red) or not containing (black) the GGUA motif in CLIP-seq (right) or RBNS (top). *P* values shown are computed by a Kolmogorov–Smirnov statistic. (**d**) RNA-binding preferences for truncated FUS as in **b**. (**e**) Scatter plot and histogram analyses are as described in **c** using FUS mouse brain CLIP-seq versus FUS RBNS *R Z*-scores. Green dots represent all significant 6mers containing the GUGG motif.

To characterize the *in vitro* sequence specificity of TAF15, we applied RNA Bind-n-Seq (RBNS)^26^ to recombinant TAF15 and, as a comparison, to recombinant FUS protein. Briefly, truncated forms of recombinant TAF15 or FUS containing both the RNA recognition motif and zinc-finger domain (amino acids 204–415 for TAF15 and amino acids 235–481 for FUS) were incubated with an RNA pool consisting of random 20mer RNAs flanked by short primers used to add adapters for high-throughput sequencing (Fig. 2a). For FUS, this truncated region was previously shown to exhibit high affinity for RNA^27^. Complementary to *in vivo* interactions identified by CLIP-seq, this method evaluates TAF15 and FUS independently of its *in vivo* complex interaction with RNA. For TAF15, RBNS discovered degenerate G-rich and GU-rich motifs and notably an (A/G)GGUA motif that resembled the GGUAAGU motif that was identified *in vivo* by CLIP (Fig. 2b). In fact, the shared GGUA 4mer was significantly enriched in hexamers that were over-represented in both RBNS and TAF15 CLIP-derived clusters relative to the appropriate control backgrounds (Fig. 2c). In addition, the same GGUA motif was enriched in the TAF15 clusters located within 3′ UTRs of target genes (Supplementary Fig. 2a). RBNS applied to FUS domains identified a similar degenerate G-rich motif, a GC-rich motif, and a GGUGG motif (bottom motif in Fig. 2d) that resembled motifs identified in published *in vivo* CLIP studies^20^,^21^. A similar evaluation of the GUGG 4mer (or GGUG, not shown) confirmed enrichment within FUS *in vivo* CLIP-seq-derived clusters in the transcriptome (Fig. 2e) and in 3′ UTRs (Supplementary Fig. 2b). Interestingly, we found that the distribution of the GUGG 4mer was also enriched in the hexamers derived from the TAF15 RBNS experiment (Supplementary Fig. 2c). Similarly, the GGUA 4mer was enriched in the FUS hexamers (Supplementary Fig. 2d). Although both of these motifs were found at a lower level of significance in the hexamers derived from experiments interrogating the other protein, our results suggest that TAF15 and FUS share some affinity with each other's motifs. It is noteworthy that the affinities of TAF15 and FUS to k-mers containing GGUA and GGUG, although significantly different from background, is relatively weak compared with previously studied RBPs such as RBFOX2, MBNL1 and CELF1 (ref. 26). We conclude that TAF15 interacts with a previously undiscovered GGUA core motif within significantly enriched clusters *in vivo*. Importantly, our results demonstrate that the interactions of FUS and TAF15 with their RNA-binding sites can occur independently of cofactor associations.

### TAF15 interacts with many FUS-RNA targets

**Figure 3:**
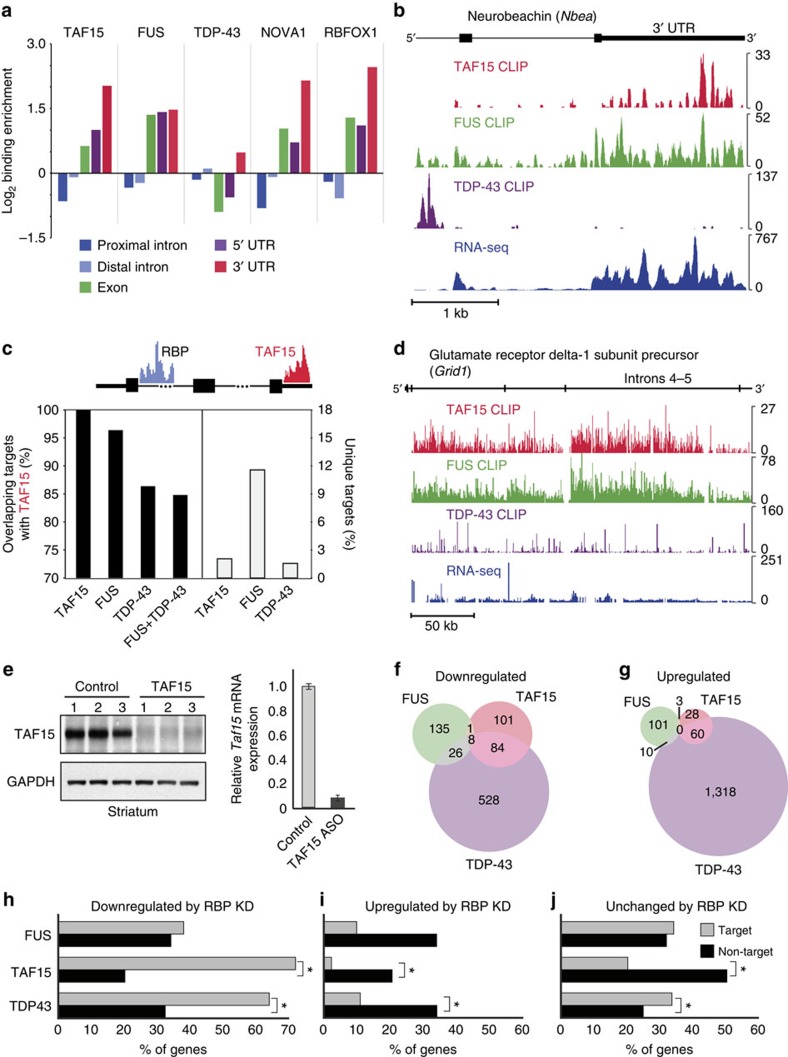
TAF15 and FUS exhibit similar RNA interaction profiles in the mouse brain. (**a**) Fold change in binding enrichment of TAF15, FUS, TDP-43, NOVA1 and RBFOX1 after normalization to the average length of proximal introns (dark blue), distal introns (light blue), exons (green), 5′ UTRs (purple) or 3′ UTRs (red). (**b**) An example of 3′ UTR binding by TAF15 (red) and FUS (green) but not by TDP-43 (purple) to Neurobeachin (*Nbea*) mRNA (chr3:55,428,730-55,433,169) in the mouse brain. RNA-seq results showing expression of *Nbea* is shown in blue. (**c**) Bar graph showing the per cent of gene targets that are common (black bars) or unique (white bars) to TAF15 and FUS, TDP-43 or both FUS and TDP-43. (**d**) An example of intronic ‘saw-tooth' binding by TAF15 (red) and FUS (green) but not by TDP-43 (purple) to the glutamate receptor delta-1 subunit precursor (*Grid1*) mRNA (chr14:35,634,350-36,071,292) in the mouse brain. RNA-seq results showing expression of *Grid1* is shown in blue. (**e**) Confirmation of reduced TAF15 expression in the mouse striatum by western blot analysis (left) and qPCR (right). Knockdown was achieved by intrastrial injection of ASOs complementary to TAF15 or a control ASO. Error bars represent s.d. (**f**,**g**) Venn diagrams showing overlap of genes downregulated (**f**) and upregulated (**g**) upon loss of TAF15, FUS or TDP-43 in the mouse striatum. (**h**–**j**) Per cent of genes that are downregulated (**h**), upregulated (**i**) or unchanged (**j**) upon ASO-mediated knockdown of the indicated RBP that has at least one binding site (target, grey) or no binding sites (non-target, black) by that RBP. Asterisks denote significant difference between target and non-target genes by Fisher's exact test at *P*<0.05.

Similar to FUS and TDP-43, TAF15 clusters were predominantly found within introns (Supplementary Fig. 3a), consistent with previously published results in HEK293 cells^28^, mouse neurons and human brain tissue^15^. As intronic regions account for a substantial proportion of nucleotides in transcribed RNA, this distribution was expected and similar to other predominantly nuclear-localized RBPs such as RBFOX1 and NOVA1 (Supplementary Fig. 3a). Unlike TDP-43, we found that TAF15 and FUS binding was significantly enriched in the 3′ UTR, akin to RBFOX1 and NOVA1 that also have proposed 3′ end formation roles^29^,^30^ (Fig. 3a). To illustrate this, the 3′ UTR of the neurobeachin (*Nbea*) transcript, encoding a protein involved in synaptic function and autism^31^, is enriched for TAF15 and FUS binding (Fig. 3b). FUS had a similar binding profile as TAF15, whereas TDP-43 bound an intronic region upstream of the penultimate exon, but with no cluster in the 3′ UTR (Fig. 3b).

We found that targets of RBFOX1 and NOVA1 do not overlap with TAF15 target genes (Supplementary Fig. 3b). In contrast, the majority of FUS (98%) and TDP-43 (86%) target RNAs were also TAF15 targets (Fig. 3c). For genes that were targets of both TAF15 and FUS, 38% had at least one binding site that overlapped between TAF15 and FUS (Supplementary Fig. 3c). Our results indicate that TAF15 and FUS bind to the same genes with close proximity, consistent with our findings that the GUGG motif preferred by FUS was enriched in TAF15 CLIP clusters and the GGUA motif preferred by TAF15 was also enriched in FUS CLIP clusters (Supplementary Fig. 2c,d). TAF15 also exhibited a ‘saw-tooth'-like pattern of deposition within genes containing long introns, such as the glutamate receptor delta-1 subunit precursor gene (*Grid1*), similar to FUS, but dissimilar to TDP-43 (ref. 21; Fig. 3d). We conclude that TAF15 and FUS binding are enriched in the 3′ UTRs of target genes and both harbour the same ‘saw-tooth'-like profiles in long introns.

### Distinct roles of TAF15, FUS and TDP-43 on gene expression

To identify TAF15-regulated RNAs, single-stranded antisense oligonucleotides (ASOs) complementary to TAF15 RNA or non-targeting control ASOs (Control) were delivered into the adult mouse striatum. TAF15 mRNA and protein were depleted by at least 90% in mice treated with TAF15-targeting ASOs (Fig. 3e). RNA extracted from striata of three mice was subjected to strand-specific RNA sequencing (RNA-seq) library generation and sequencing. On average, 24.8 million reads were obtained for each library, of which 86% mapped to the mouse genome (mm9). We identified 194 and 91 genes (Supplementary Data 1) that were significantly (*P*<0.05) downregulated (Fig. 3f) and upregulated (Fig. 3g), respectively, when TAF15 protein was depleted. To examine overlapping and unique effects of TAF15, FUS and TDP-43 on RNA expression, we re-analysed RNA-seq data sets in which FUS and TDP-43 were depleted from the mouse striatum in the same manner as TAF15 (refs 21, 22). Although *Fus* and *Tdp-43* expression remained unchanged upon TAF15 depletion, the *Taf15* mRNA level appears to be slightly increased upon FUS depletion (Supplementary Fig. 3d). Similar to FUS and TDP-43 (ref. 21), we found that genes downregulated by loss of TAF15 exhibited exceptionally long introns (Supplementary Fig. 3e). Despite this similar trend in regulation, there was a poor overlap between the differentially regulated genes such that by our conservative re-analysis, only eight genes (including *Park2*, *Nrxn1* and *Kcnip4*) were commonly downregulated (Fig. 3f) and no genes were commonly upregulated (Fig. 3g). To distinguish between direct and indirect effects of RBP binding on gene expression, we measured the fraction of affected genes that were directly bound as determined by CLIP-seq. A significantly higher proportion of genes downregulated upon TAF15 or TDP-43 loss were direct targets of that RBP (Fig. 3h). Closer examination revealed that this association remains significant for the subset of downregulated genes that exhibited TAF15 (and to some extent TDP-43) binding in the 3′ UTR (Supplementary Fig. 3f). In support of this result, genes that were downregulated upon TAF15 loss were more likely to contain the TAF15 ‘GGUAA' motif in their 3′ UTRs or introns (Supplementary Fig. 3g). Genes that were upregulated or were unaffected upon loss of FUS, TAF15 or TDP-43 were generally not binding targets (Fig. 3i,j). Thus, we conclude that, although FUS, TAF15 and TDP-43 bind many of the same targets, only a small fraction of genes are similarly affected by loss of each of the three RBPs.

### TAF15 has a marginal role in AS

**Figure 4:**
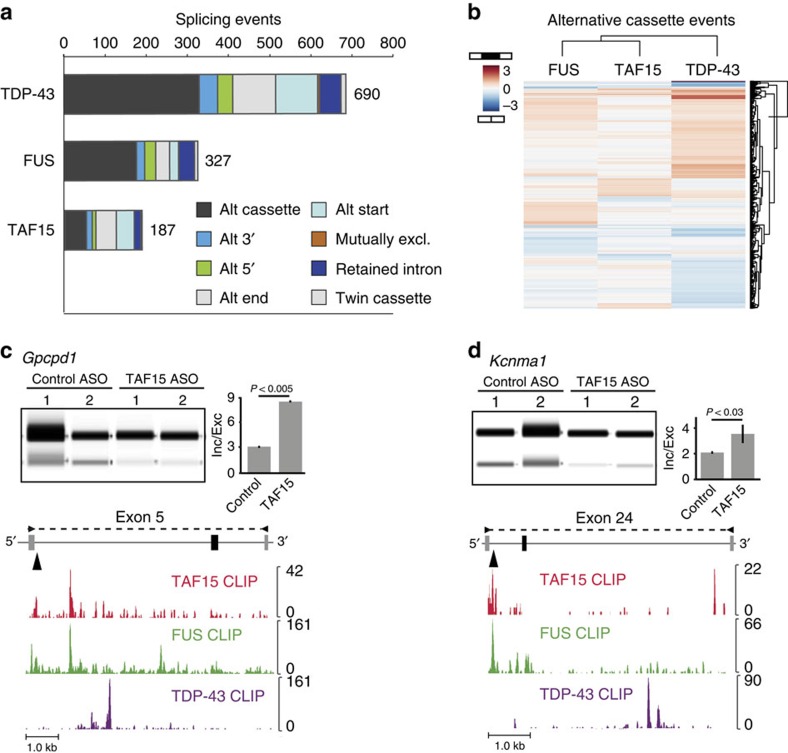
TAF15 influences alternative splicing for a small subset of transcripts. (**a**) Bar graph showing the number of alternative splicing events altered upon ASO-mediated depletion of TDP-43, FUS or TAF15 in the mouse striatum, as detected by splicing-sensitive microarray analyses. (**b**) Heatmap of alternative cassette events in **a** altered by FUS, TAF15 or TDP-43 depletion. Hierarchical clustering analysis was performed using separation (Sep) scores. Higher Sep scores (red) indicate inclusion events and lower Sep scores (blue) indicate exclusion events. (**c**) RT–PCR for exon 5 of glycerophosphocholine phosphodiesterase 1 (*Gpcpd1*; chr2:132,382,646-132,390,412) to assess alternative splicing in TAF15 knockdown samples compared with controls. Quantification of biological replicates is shown. Error bars represent s.d. Binding of TAF15 (red), FUS (green) and TDP-43 (purple) in the mouse brain is shown below. (**d**) RT–PCR of exon 24 in the potassium channel, calcium-activated large conductance subfamily M alpha, member 1 (*Kcnma1*) (chr14:24,149,961-24,156,401) to assess alternative splicing in TAF15 knockdown samples compared with controls. Quantification of biological replicates is shown. Error bars represent s.d. Binding of TAF15 (red), FUS (green) and TDP-43 (purple) in the mouse brain is shown below.

Using splicing-sensitive microarrays, we detected 182 AS events that were altered upon TAF15 depletion in the mouse striatum (Fig. 4a). Although TAF15, TDP-43 and FUS proteins were reduced to similar levels, we observed fewer TAF15-dependent splicing events (*n*=187) compared with the number of splicing events altered by loss of FUS (*n*=327) or TDP-43 (*n*=690; Supplementary Data 2). There was little overlap in AS events altered by loss of TAF15, FUS or TDP-43 (Fig. 4b and Supplementary Fig. 4a). This suggests that, despite their high similarity in domain architecture and documented interactions with splicing factors^32^,^33^, FUS and TAF15 have distinct impacts on AS.

An AS event altered by TAF15 is exon 5 of the glycerophosphocholine phosphodiesterase 1 gene (*GPCPD1*; Fig. 4c), which was included upon TAF15 knockdown and harbours binding sites for TAF15 and FUS downstream of the 5′ flanking exon (indicated by an arrow). Another example, exon 24 in the calcium-activated potassium channel subunit alpha-1 gene (*Kcnma1*; Fig. 4d), was also included upon TAF15 loss and contained nearby TAF15- and FUS-binding sites (indicated by an arrow). Although TDP-43-binding sites were present near these exons, they were distinct from TAF15- and FUS-binding sites (Fig. 4c,d). A previous study reported that knockdown of TAF15 promoted the exclusion of exon 19 of the N-Methyl-D-Aspartate Receptor Subunit NR1 (*Grin1*) gene in mouse neurons^15^. TAF15- and FUS (but not TDP-43)-binding sites were present proximal to this exon, but depletion of TAF15 did not cause a statistically significant change (*P*=0.106) in the exclusion of exon 19 of *Grin1* in the mouse striatum (Supplementary Fig. 4b). This does not appear to be because of differences in tissue specificity as depletion of TAF15 in the mouse brain or spinal cord (Supplementary Fig. 4c) also had no significant effect on *Grin1* exon 19 splicing (Supplementary Fig. 4d). We conclude that in contrast to our and others' previous findings with FUS and TDP-43 (refs 20, 21, 22, 34) and a study regarding TAF15 (ref. 15), TAF15 alters the splicing of a relatively small subset of genes, of which the majority (70%) are distinct from those regulated by either FUS or TDP-43.

### TAF15 and FUS affect mRNA stability in neural progenitors

**Figure 5:**
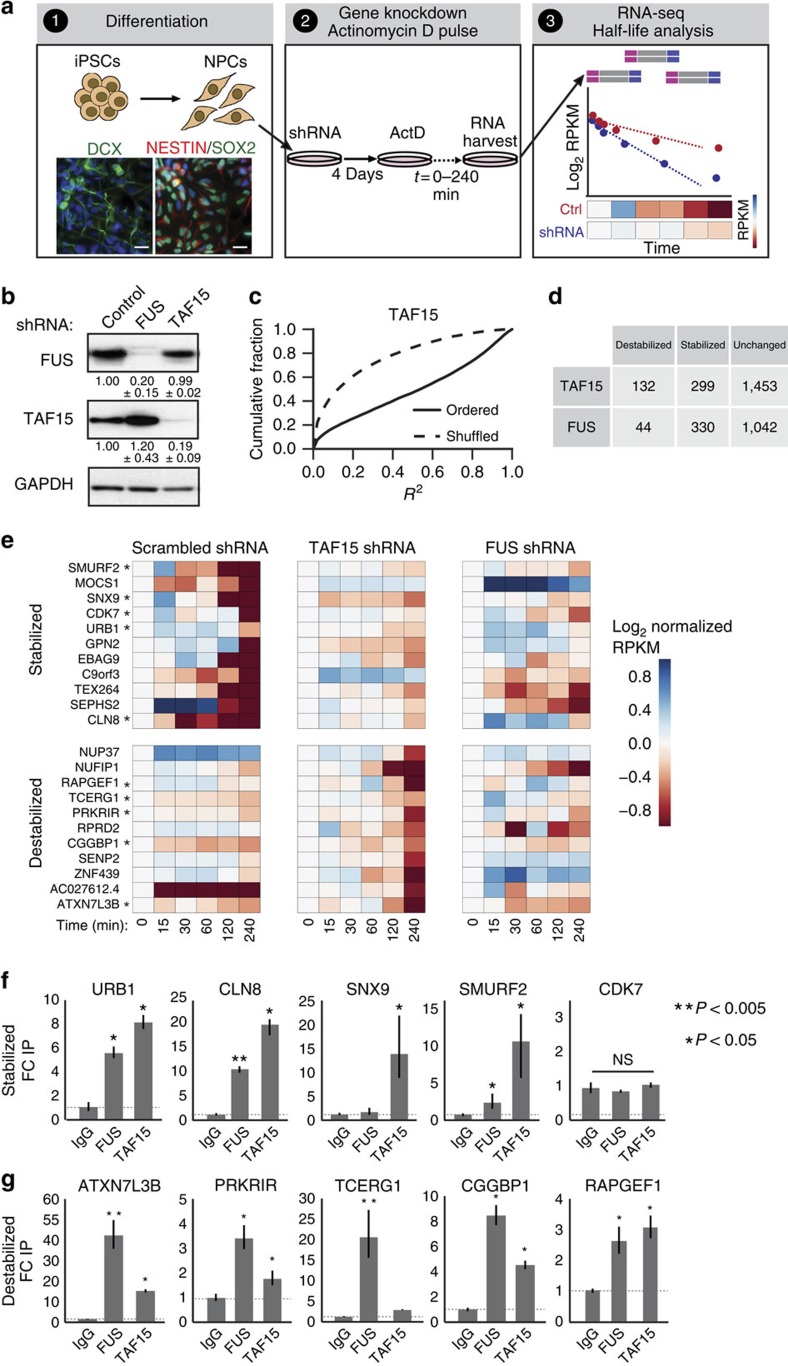
Loss of TAF15 or FUS affects mRNA stability in human neural precursor cells. (**a**) Schematic of workflow. (1) Human iPSCs were differentiated into NPCs and stained for neuronal lineage markers DCX (left, green), NESTIN (right, red) and SOX2 (right, green) to confirm differentiation. 4,6-Diamidino-2-phenylindole (DAPI; blue) was used to locate nuclei. Scale bar, 25 μm. (2) NPCs were infected with virus expressing shRNAs against TAF15 or FUS and then treated with Actinomycin D for indicated duration. (3) Poly(A)-selected RNA was converted into libraries for sequencing, and sequencing reads were used to calculate mRNA half-lives. (**b**) Validation of shRNA-mediated knockdown of FUS or TAF15 in NPCs by western blot analysis. Representative western blot is shown from one replicate with quantifications from biological triplicate knockdown experiments. (**c**) For each gene, the coefficient of determination (*R*^2^) reflecting the fit of the RPKM values to a log-linear regression was computed. The cumulative distribution functions of the *R*^2^ values for all genes in the TAF15 depletion experiment are depicted for real and shuffled values. (**d**) Table displaying the number of mRNAs whose half-lives were destabilized, stabilized or unchanged by depletion of TAF15 or FUS. Half-life changes, measured as log_2_ (knockdown/control), that were greater than 1 were considered. (**e**) Heatmap of normalized RPKMs for stabilized and destabilized mRNAs upon shRNA-mediated knockdown of TAF15 or FUS. RPKMs are normalized for each gene to its RPKM at time 0. An asterisk indicates that the gene was examined for binding in **f**. (**f**) RNA immunoprecipitation was performed using antibodies against IgG (Control), TAF15 and FUS in NPCs. The relative fold change compared with the IgG control for genes that are stabilized by TAF15 loss was determined with qPCR. Values are means±s.d. for biological duplicates. Asterisk denotes a significant difference compared with IgG by Student's *t*-test where **P<0.005 and *P<0.05. (**g**) RNA immunoprecipitation analysis as in **f** for mRNAs that were destabilized upon TAF15 loss.

To evaluate the role of TAF15 in early neuronal development, we used human NPCs differentiated from iPSCs in which TAF15 protein levels and, for comparison, FUS protein levels, were individually depleted by lentiviral short hairpin RNAs (shRNAs; Fig. 5a,b). As TAF15 has a relatively minor role in AS, we investigated a potential role for TAF15 in RNA stability. NPCs were treated with the transcriptional inhibitor Actinomycin D for varying times, after which total RNA was collected and prepared for RNA-seq libraries (Fig. 5a). Half-life measurements were determined from a regression-based analysis of gene expression, assuming first-order decay kinetics. For each shRNA treatment, the median value for the coefficient of determination (*R*^2^) describing the log-linear fit across the time course for each gene, across all genes, was 0.54; this value was significantly higher (*P*∼0, by Kolgomorov–Smirnov two-tailed test) than the value (0.12) obtained by randomly shuffling the expression values for each time point within each gene (Fig. 5c and Supplementary Fig. 5a,b). To minimize false positives, we evaluated genes for which the *R*^2^ value was greater than 0.6. We identified 299 and 330 genes that were highly stabilized (increased half-life), as well as 132 and 44 genes that were highly destabilized (decreased half-life) upon loss of TAF15 and FUS, respectively (Fig. 5d, Supplementary Data 3).

We arbitrarily selected mRNAs that were most stabilized or destabilized by loss of TAF15 (marked by asterisks in Fig. 5e) and performed RNA immunoprecipitation followed by quantitative reverse transcriptase PCR (qRT–PCR) to determine whether these mRNAs were directly bound by TAF15 and FUS. TAF15 bound to most mRNAs (four of five) that were stabilized upon loss of TAF15 (*URB1*, *SNX9*, *CLN8* and *SMURF2*; Fig. 5f), of which *URB1*, *CLN8* and *SMURF2* also exhibited FUS interactions. In addition, TAF15 bound to most mRNAs (four of five) that were destabilized upon loss of TAF15 (*ATXN7L3B*, *PRKRIR*, *RAPGEF1* and *CGGBP1*; Fig. 5g). Notably, FUS bound to all these mRNAs (including *TCERG1*); however, FUS depletion did not appear to have an effect on mRNA stability of these transcripts (Fig. 5e and Supplementary Data 4). *ANAX2* and *TIAL1*, whose mRNAs were unaltered by TAF15 loss, were also examined for TAF15 and FUS binding (Supplementary Fig. 5c). A gene ontology (GO) analysis of genes affected at the mRNA stability level upon TAF15 knockdown revealed statistical enrichment for genes implicated in DNA-dependent transcription control (*P*<10^−26^; Supplementary Data 5). An example of TAF15 mRNA turnover target involved in transcriptional control and also neurological diseases is the CGG-binding protein 1 (*CGGBP1*), which binds to CGG repeats in the promoter of the fragile X mental retardation 1 (*FMR1*) gene resulting in reduced expression^35^. We conclude that TAF15 and FUS control mRNA turnover in NPCs of distinct mRNA substrates.

### TAF15 and FUS affect different genes in human MNs

**Figure 6:**
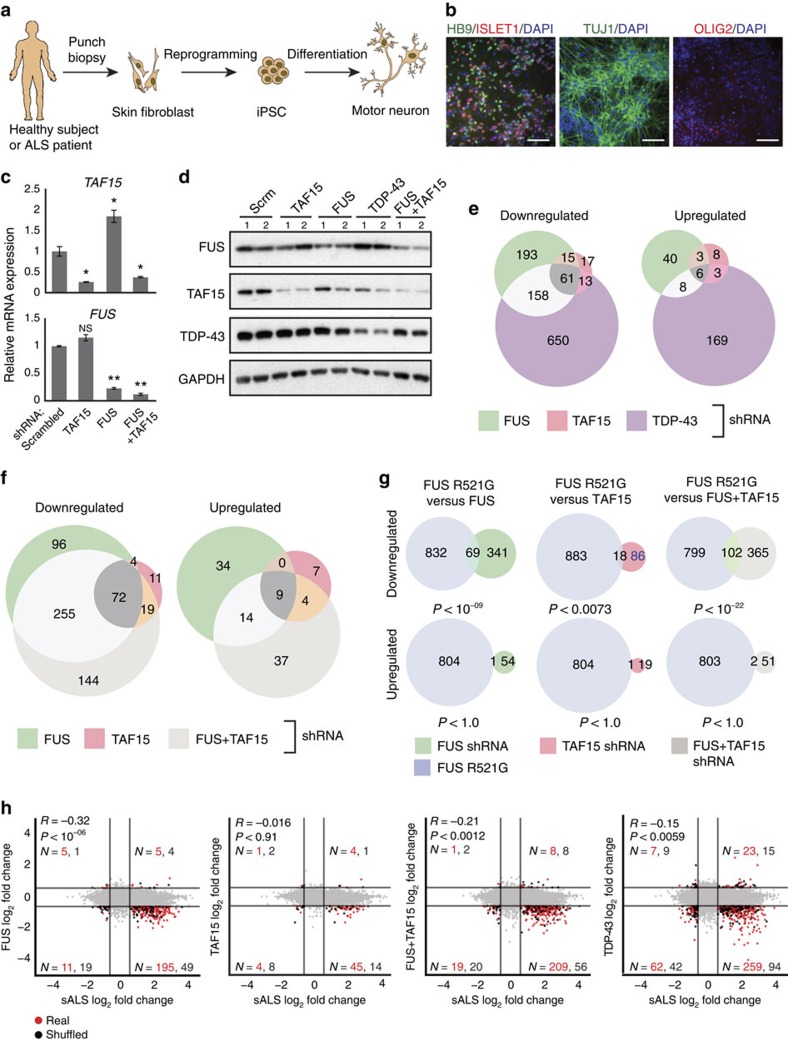
Comparison of MN RNA signatures upon TAF15, FUS or TDP-43 loss to two models of ALS. (**a**) Schematic of workflow to reprogramme iPSCs and to differentiate into MNs. (**b**) Immunofluorescence of human iPSC-derived MNs for MN marker HB9 (green), post-mitotic neuronal marker TUJ1 (green), neural stem cell marker ISLET1 (red) and oligodendrocyte marker OLIG2 (red). DAPI stain marks cell nuclei (blue). Scale bar, 25 μm. (**c**) qRT–PCR and (**d**) western blot validation of shRNA-mediated depletion of TAF15, FUS and TDP-43 in MNs. Error bars represent s.d. from biological duplicate experiments. (**e**,**f**) Venn diagrams showing overlap of up- and downregulated genes in MNs upon depletion of TAF15, TDP-43, FUS or, simultaneously, FUS and TAF15 (FUS+TAF15). (**g**) Venn diagrams showing overlap of up- and downregulated genes between MNs with the FUS R521G mutation and knockdown of TAF15, FUS or FUS+TAF15. Statistical significance was determined by a hypergeometric test using genes expressed in MNs as background. (**h**) Scatter plots comparing gene expression changes (log_2_ RPKM) in MNs from sALS patient samples compared with loss of TAF15, FUS, TDP-43 or FUS+TAF15. Each quadrant of a scatter plot shows genes (red dots) and gene counts (*N*, in red) that are significantly changing in sALS and RBP depletion experiments. Genes from a randomly ordered comparison are also shown (black dots) along with gene counts (*N*, in black). *R*^2^ and *P* values from linear regression analyses of genes significantly changing in both sALS and RBP knockdown experiments are shown.

To discover the molecular events modulated by loss of TAF15, FUS and TDP-43 in an ALS-relevant cell type, we generated MNs from wild-type human iPSCs using a directed differentiation protocol^36^. Briefly, a combination of SMAD signalling inhibitors, Noggin, and the ALK5 inhibitor, SB431542, was used to yield a population of cells enriched for HB9, ISLET1 and TUJ1 (neuron-specific class III)-positive MNs with a minor fraction of OLIG2-positive oligodendrocytes (Fig. 6a,b). We subjected the MNs to lentivirus-packaged shRNAs targeting TAF15, FUS or TDP-43. As our *in vivo* findings indicated that TAF15 and FUS bind to similar RNA substrates, we also simultaneously depleted FUS and TAF15. Mature RNA and protein levels (Fig. 6c,d and Supplementary Fig. 6a) of the targeted RBPs were significantly reduced and TAF15 and FUS protein levels did not exhibit reproducible changes (either up or down) in FUS and TAF15 depletions, respectively (Supplementary Fig. 6a). Reduction of TAF15, FUS or TDP-43 alone or in combination (TAF15 and FUS) in iPSC-derived MNs did not cause noticeable changes in cell morphology or death. We generated RNA-seq data from these cells, obtaining an average of 32.4 million uniquely mapped reads.

Similar to our *in vivo* depletion studies, we observed a minor overlap in the genes downregulated (61 genes) or upregulated (6 genes) upon loss of all three RBPs (Fig. 6e and Supplementary Data 6). In contrast to our findings in the adult mouse striatum, introns within downregulated genes affected by loss of TAF15 and FUS in MNs were not significantly longer than upregulated or unaffected genes (data not shown). Expectedly, ∼76 and 85% of the genes in the FUS-only and TAF15-only knockdown experiments were also downregulated in the double knockdown. However, we found that a subset of genes (*n*=144) were downregulated only upon combined loss of TAF15 and FUS in human MNs (Fig. 6f), indicating a potential redundancy between TAF15 and FUS in controlling gene expression. These genes that were downregulated upon combined TAF15 and FUS loss were enriched for GO terms, reflecting extracellular cellular matrix composition, cell proliferation, wound healing and cytokine activity.

### Genes altered by RBP loss are akin to ALS-linked FUS mutant

To investigate whether the molecular changes observed upon loss of FUS, TAF15 or both proteins were relevant to ALS pathogenesis, we obtained fibroblasts from two ALS patients with the causative R521G mutation in *FUS*. The fibroblasts were reprogrammed into iPSCs and subjected to cellular, molecular and genetic characterization to confirm that they are pluripotent (Supplementary Fig. 6b), exhibit a normal karyotype and harbour the presence or absence of the mutation at nucleotide position 1,561 (Supplementary Fig. 6c). Three individual clones from two FUS R521G patient-derived iPSC lines (two clones were from one line) and two control iPSCs (from healthy, age-matched non-mutant individuals) were directly differentiated to MNs. RNA isolated from these cells were subjected to RNA-seq library preparation and sequencing to obtain an average of 20 million reads, of which 90% mapped uniquely to the human genome (hg19). To ensure that the differentiation process yielded MNs at similar stages of differentiation and similar subtypes of cells, we compared expression of a panel consisting of genes representing housekeeping, astrocyte, oligodendrocytes, neural precursor and neuronal subtypes. The similarities in expression profiles among the MN cell lines confirmed that differentiation of the iPSC lines was consistent and hence enabled downstream comparative analysis (Supplementary Fig. 6d). We identified 901 downregulated and 805 upregulated (Supplementary Data 7) genes that were differentially affected in the FUS R521G MNs compared with wild-type control MNs. Interestingly, although the majority of mutant-dependent gene expression changes were unique, there existed statistically significant overlaps in the genes downregulated in the FUS R521G MNs (relative to control) with genes downregulated upon loss of FUS (*P*<10^−9^) or TAF15 (*P*<10^−3^; Fig. 6g). Importantly, this overlap increased in number when we compared the genes affected by simultaneous depletion of both FUS and TAF15 (*P*<10^−22^; Fig. 6g). In contrast, we observed no significant overlap in genes upregulated by any condition (Fig. 6g). Overall, these findings are consistent with our observations that FUS and TAF15 are redundant in their effects on molecular targets and implies a partial loss of molecular function by the FUS R521G mutation.

### Downregulated genes correlate with a sALS RNA signature

To obtain insights into whether the genes affected by loss of ALS-associated RBPs resemble disease-specific RNA signatures, we turned to a RNA-seq data set generated from laser-capture microdissected spinal cord samples from sALS patients who had bulbar or arm onset of disease that was caudally progressing and thus had abundant residual MNs in the lumbar region at the time of death^37^. The RNA-seq data set consisted of samples from 13 sALS and 9 control patients. In all, 3,876 genes were significantly differentially regulated, of which 71% and 29% were upregulated and downregulated, respectively, in the sALS patient compared with normal samples (Supplementary Data 8). The differentially expressed genes were effectively able to separate the diseased patients from the control patients (Supplementary Fig. 6e). Next, we tested the hypothesis that ALS RBP-mediated RNA changes resemble the RNA signature that distinguishes sALS and normal samples. For all the comparisons performed, we observed a significant overlap (*P*<0.05, hypergeometric test) in genes that were upregulated in sALS samples and downregulated in FUS, TAF15, TDP-43, or FUS and TAF15 double knockdowns (Supplementary Fig. 6f). We also observed a significant inverse correlation of significantly changing genes between sALS samples and FUS, TDP-43 or FUS and TAF15 double knockdowns (Fig. 6h, *R*^2^ between −0.14 and −0.32, *P*<0.05), but not between sALS samples and the FUS R521G mutant MNs (Supplementary Fig. 6g). Despite the divergent sets of regulated genes whose mRNA levels are dependent on ALS-associated RBPs, we found that 2,747 genes were upregulated in sALS patient samples. Unlike *in vitro* differentiated MNs, the sALS patient samples represent more mature MNs at a late stage of disease progression. Our findings indicate that in late-stage sALS patient samples with TDP-43 pathology^37^, a subset of genes that are separable from those found in ALS iPSC-derived FUS R521G MNs, are abnormally higher compared with control patients. Among the commonly differentially regulated genes between knockdown and sALS samples, GO terms for extracellular space and matrix organization were statistically enriched (*P*<0.01).

## Discussion

Genetic and clinical evidence strongly supports causative roles for FUS, TDP-43 and TAF15 in ALS. Here we identify common and unique pathways normally controlled by these proteins utilizing diverse *in vitro* and *in vivo* neuronal systems (Supplementary Fig. 7). In the adult mouse brain, we identified TAF15-binding sites within ∼4,900 RNA substrates, and a GGUAAGU TAF15-binding motif not reported in previous studies^15^,^28^. We used RBNS technology to confirm a GGUA motif that was enriched within *in vivo* TAF15-binding sites. Together, we conclude that TAF15 and FUS can interact with their RNA motifs within *in vivo* RNA substrates without requiring complex cofactor associations. Overall, the RNA-binding pattern of TAF15 resembled that of FUS, but was distinct from TDP-43, even when all three RBPs targeted the same genes. TAF15 and FUS exhibited saw-tooth-like binding patterns on long introns, a pattern reminiscent of co-transcriptional splicing^38^. Genes downregulated upon loss of either TAF15 or FUS contained exceptionally longer introns. In addition, TAF15- and FUS-binding sites were also over-represented within 3′ UTRs, possibly reflecting 3′ end processing functions such as RNA turnover, transport and translation. Upregulation of genes upon FUS and TAF15 loss is likely a secondary effect as these genes are generally not targets. Lastly, unlike TDP-43 and FUS, loss of TAF15 appeared to have a minor impact on AS in the adult mouse brain.

In models of early human neuronal development, we identified that TAF15 and FUS affected the mRNA turnover of distinct subsets of RNA targets in human neuronal progenitor cells. Furthermore, loss of FUS, TAF15 or TDP-43 in human MNs derived from the same cells resulted in distinct changes in gene expression for each RBP. In addition, simultaneous depletion of FUS and TAF15 resulted in the downregulation of hundreds of additional genes. FUS and TAF15 have been shown to interact with each other^23^,^24^ (Supplementary Fig. 1b) as well as other common proteins such as RNA Pol II (refs 39, 40), spliceosome machinery^24^,^32^,^33^ and transcription factors^41^. One possibility is that if FUS is unable to recruit regulatory factors to an RNA target, this function may be compensated for by TAF15.

To gain insight into disease, we compared the results of our loss-of-function studies to two models of ALS. The first model is MNs from ALS patients carrying the pathogenic *FUS R521G* mutation. Expression of *FUS R521G* from the mouse *MAPT* locus has been recently reported to cause neuronal toxicity in neurons of mice^42^. Previously, FUS R521G was also associated with a partial loss-of-function in RNA regulation in mouse spinal cords^43^. We did observe a small yet significant overlap in genes downregulated upon loss of TAF15, FUS or both proteins, and genes altered by FUS R521G. This overlapping set of genes may reflect the partial loss-of-function properties of FUS R521G (ref. 43). As mRNAs downregulated upon loss of these RBPs in the mouse brain are often direct binding targets of those RBPs, we speculate that the *FUS R521G* mutation, which causes cytoplasmic FUS mislocalization, resembles a partial loss-of-function of the RBPs in a model of early development. Nevertheless, the majority of expression changes caused by FUS R521G were mutant-specific such that they did not overlap with genes altered by loss of TAF15, FUS or both proteins. One interpretation is that these FUS R521G-specific gene changes may contribute the pathological, gain-of-function activities of mutant FUS that was observed to cause MN dysfunction in mice^42^.

To model late-stage ALS disease we utilized RNA-seq data obtained from spinal cord samples collected postmortem by laser-capture microdissection from sALS patients. These samples harboured ubiquinated TDP-43 cytoplasmic inclusions and were from patients with no mutations in known ALS-causative genes, including *FUS*, *TDP-43* or *TAF15*. Intriguingly, our comparisons of RNA signatures revealed an inverse correlation in a separate set of genes that were upregulated in the sALS samples but were downregulated upon loss of ALS-associated RBPs in *in vitro*-derived MNs. This indicates that these genes, whose levels are normally dependent and maintained by FUS, TAF15 and TDP-43, are aberrantly higher in late-stage ALS. A possible mechanism for gene upregulation is the breakdown of negative feedback loops as is observed for the effect of TDP-43 on its own expression^44^,^45^. We did not, however, observe a difference in TDP-43 mRNA levels between sALS and control neurons. Another plausible scenario is that in late stages of the disease, cytoplasmic inclusions of TDP-43 lead to stabilization of trapped, cytoplasmic RNA targets. Future studies to identify the mislocalized RNA targets in cytoplasmic bodies that are protected from degradation, such as stress granules, may yield further insight into disease-relevant targets at late stages in the disease.

In summary, our study delineates convergent and divergent RNA-processing functions of ALS-associated FUS, TAF15 and TDP-43 in normal and disease settings. Our comprehensive results shed light on multiple and distinct pathways by which these RBPs regulate gene expression in diverse neuronal systems and provide a framework for how they relate to ALS and other neurodegenerative diseases.

## Methods

### Injections of ASO in mice

Sterotaxtic injections of ASO complementary to TAF15 were performed in 8-week-old female C57Bl/b mice to deplete TAF15. ASOs were delivered specifically to the striatum or brain/spinal cord by intrastriatal (12.5 μg) or intracerebroventricular injection (300 μg), respectively, as described previously^21^,^46^. Female mice were regularly monitored for 14 days until being killed and the tissues were harvested and frozen in TRIzol (Invitrogen). Control mice received a control ASO without any known target in the mouse genome under the same conditions. The ASOs were phosphorothioate ‘gapmers' with sequences as follows (capitalized nucleotides containing 2±-O-(2-methoxy)ethyl modifications): GGTCTcctccatagcTGCCT (TAF15; brain and striatum), TGGCAatattttacaACGCA (TAF15; spinal cord) and CTCAGTAACATTGACACCAC (Control). All procedures were performed using a protocol approved by the Institutional Animal Care and Use Committee of Ionis Pharmaceuticals and the University of California at San Diego.

### Generation of neural precursor cells and MNs

Human iPSCs derived from dermal fibroblast cells of a healthy individual (RRN08) were induced into neural precursor cells using a pan-neuronal protocol as previously described^21^. Briefly, stem cells were grown on Matrigel-coated plates (BD) in mTeSR1 growth media (Stem Cell Technologies). Stem cell colonies were grown on ultra low-attachment plates in DMEM/F12 + GlutaMAX supplemented with N2 and FGF-2 (20 ng ml^−1^). After 1 week, neural rosettes were manually picked, replated and maintained in DMEM/F12 + GlutaMAX supplemented with N2, B27 and FGF-2 (20 ng ml^−1^).

### Generation of human MNs

Human MNs used in the shRNA knockdown experiments were differentiated from iPSCs (CVB) using a protocol modified from (ref. 36). Briefly, human iPSCs were maintained in hEB Media (Knockout D-MEM + 10% Knockout Serum Replacement (Life Technologies) + 10% Plasmanate (Biocare) + GlutaMAX + NEAA (Life Technologies) and supplemented with 10 μM SB431542 and 1 μM Dorsomorphin dihydrochloride (Tocris) on feeder-free dishes. Cells were maintained in SB431542 and Dorsomorphin until day 18 of differentiation. On days 4, 5 and 6 of differentiation, hEB media were mixed with N2 Base media (D-MEM/F12 + GlutaMAX, 1% N2 Supplement + 4.5 mM D-Glucose, 0.05 mM Ascorbic Acid (Sigma)) at a ratio of 70:30, 50:50 and 50:50, respectively. On days 7 and 8 of differentiation, cells were maintained in 50:50 combination of hEB media and maturation media (D-MEM/F12 + GlutaMAX, 2% N2 Supplement, 4% B27 serum-free supplement (Invitrogen), 9.0 mM D-Glucose and 0.1 mM ascorbic acid (Sigma)) supplemented with 2 ng ml^−1^ each of ciliary neurotrophic factor, brain-derived neurotrophic factor and glial cell-derived neurotrophic factor (Peprotech). From days 7 to 22 of differentiation, cells were treated with 200 nM Smoothened Agonist (SAG; EMD Biosciences) and 1.5 μM retinoic acid (Sigma). On day 18, cells were dissociated using Accutase and transferred to dishes coated with Poly-D-Lysine (Sigma) + Laminin (Life Technologies) and maintained in maturation media supplemented with retinoic acid and SAG. On day 22, cells were maintained in maturation media containing 2 μM DAPT (Tocris). On day 26, cells were maintained in maturation media only. Throughout the differentiation protocol media was changed daily. The identity and purity of MNs were analysed by immunofluorescence for markers of stem cells, MNs, astrocytes and glial cells.

### Generation of MNs from fibroblast-derived iPSCs

Adult human primary fibroblasts were obtained by Franca Cambia, Edward Kasarskis and Haining Zhu (University of Kentucky). Informed consent was obtained from all subjects before sample collection. The use of patient fibroblasts for research was approved by the University of Kentucky Institutional Review Board (IRB 05-0265). Briefly, adult human primary fibroblasts were cultured at 37 °C and 5% CO_2_ in DMEM supplemented with 10% fetal bovine serum (FBS), NEAA and L-glutamine. To generate iPS cells, control and ALS patient fibroblasts were transduced with the CytoTune iPS Sendai Reprogramming Kit, as described in the manufacturer's protocol (Invitrogen). Colonies were manually passaged on Matrigel-coated plates and grown in mTeSR1 growth media. After several passages, colonies were expanded using Accutase (Innovative Cell Technologies) and grown as a monolayer before differentiation. MN differentiation was performed as described above with the following modifications: CHIR99021 (Tocris) was added at 4 μm until day 7 and the cells were either fixed for immunostaining or harvested for RNA in TRIzol (Life Technologies) 35 days post-neural induction. Three ALS patient lines GY6.2, GY7.3 and GY7.6 are referred to as FUS R521G Line 1, Line 2 and Line 3, respectively, and two wild-type sibling control lines KIN1ALS17.3 and KIN1ALS17.4 are referred to as wild-type sibling control Line 1 and Line 2, respectively.

### Lentiviral infections and transfections

Lentiviral shRNA constructs (Open Biosystems) complementary to human *TAF15* (TRCN0000020140, TRCN0000020141 or TRCN0000020143), human *FUS/TLS* (TRCN0000010450, TRCN0000039824 or TRCN0000039825) and human *TDP-43* (TRCN0000016038) in the pLKO.1 vector system were used to produce lentivirus as previously described^47^. Virus produced from a pLKO.1 construct containing a control sequence was used as the control. At 60–70% confluency, NPCs were infected with virus (multiplicity of infection=3) for 24 h, followed by a complete media change and further incubation for 72 h until cells were either collected and frozen in TRIzol (Invitrogen) or pelleted and frozen in liquid nitrogen for RNA and protein analyses, respectively. For lentiviral infection of MNs, media containing virus (multiplicity of infection=5) were added to cells on day 28 of the MN differentiation protocol. After 24 h, a complete media change was performed and cells incubated for an additional 48 h. A second round of infection, similar to the first, began on day 31. On day 34 of MN differentiation, corresponding to a 6-day exposure period to shRNA expression, cells were either collected and frozen in TRIzol (Invitrogen) or pelleted and frozen in liquid nitrogen for RNA and protein analyses, respectively. For transfection of HEK293T cells, cells were plated in DMEM high-glucose media (Life Technologies) supplemented with 10% FBS. Cells were transfected with plasmid expressing human FUS-Myc cloned into pcDNA5 or TAF15-V5 cloned into pEF5-DEST using FuGENE 6 (Promega) according to the manufacturer's protocol for 24 h and then harvested for protein analysis.

### CLIP-seq library preparation and sequencing

Brains from 8-week-old female C57Bl/6 mice were rapidly dissociated by forcing the tissue through a cell strainer with a pore size of 100 μm (BD Falcon) before ultraviolet crosslinking. CLIP-seq libraries were constructed as previously described^47^ using 10 μg of a polyclonal antibody against TAF15 (300A-308, Bethyl Laboratories). Libraries were subjected to sequencing on a HiSeq2000 platform for 50 cycles. For each CLIP-seq library, the brain of one mouse was used.

### Computational analysis of CLIP-seq experiments

CLIP-seq alignment and peak calling were performed as previously described^21^. Briefly, reads with the sequencing adapter or homopolymeric runs were trimmed and then mapped to the repeat-masked mouse genome (mm9) using Bowtie (version 0.12.2) with parameters −q −p 4 −e 100 −a −m 10 −best–strata. Reads that were flagged as PCR duplicates were removed. Significant clusters of reads were identified using a Poisson distribution with two different frequencies to determine a *P* value. First, a transcriptome-wide frequency was calculated by dividing the total length of all pre-mRNAs by the total number of CLIP reads mapping to the whole pre-mRNA transcriptome. Second, a gene-specific frequency was calculated by dividing the size of the gene-specific pre-mRNA by the total number of CLIP reads mapping to that gene-specific pre-mRNA. A significant cluster was annotated if it had sufficient reads to exceed a Bonferroni-corrected *P*<10e^−4^ using both frequencies against the Poisson distribution

### *De novo* motif analysis

Motif analysis was performed as previously described^48^. Briefly, HOMER^49^ was used to call *de novo* motifs using the command ‘findMotifs.pl <foreground> fasta <outloc> -nofacts –p 4 –rna –S 20 –len 5,6,7,8,9 –noconvert –nogo –fasta <background>. Foreground is defined as a fasta file of sequences taken from all called clusters, or all called clusters in a specific transcriptome region and background was randomly located clusters within the same genic regions as predicted TAF15 clusters.

### Peak annotations

Transcriptome regions and gene classes were defined using annotations found in GENCODE version 17 (ref. 50). Depending on the analysis, clusters were either associated by the GENCODE-annotated 5′ UTR, 3′ UTR, exon or intronic regions. If a cluster overlapped multiple regions or a single part of a transcript was annotated as multiple regions, clusters were iteratively assigned first as exon, then 3′ UTR, 5′ UTR and finally as proximal or distal introns (as defined as 500 bp or greater from an exon–intron boundary). Overlapping peaks were calculated using bedtools^51^,^52^.

### Enrichment of peaks relative to region size

To compute the fold enrichment of peaks in a given region, the fraction of peaks in that region was calculated as described above. The fractional region size was derived by dividing the total number of base pairs in that region relative to the total number of base pairs in all regions. Fold enrichment was computed using the equation log_2_ (*F*_CLIP_/*F*_region_).

### Distance of peaks from motifs

Distance from peaks was computed by using the annotatePeaks function in HOMER^49^ with the arguments ‘annotatePeaks.pl <peaks> mm9 -m <motif>, -hist 10-size 1000 –noann'. Identification of peaks and motifs was determined as described above.

### RBNS

RBNS was performed as previously described^26^. Briefly, truncated reading frames of FUS (amino acids 204–415) and TAF15 (amino acids 235–418), which contain all RNA-binding domains, were cloned downstream of a tandem GST-SBP tag into a modified pGex6p-1 vector (GE). Truncated proteins were recombinantly expressed and purified via the GST tag, and used for RBNS, which was performed at 5 concentrations (0, 5, 20, 80 and 320 nM) with a pool of randomized 20mer RNAs, flanked by short primers. Preparation of the randomized RNA pool and all reaction conditions was identical to previous descriptions^26^. Further computational analysis details can be found in Supplementary Methods.

### RNA-seq library preparation and analysis

Total RNA was extracted from mouse tissues and human cells using TRIzol (Invitrogen) according to the manufacturer's instructions. Total RNA (0.5–3 μg) was DNase-treated and subjected to poly(A) selection or Ribo-Zero treatment followed by library preparation using TruSeq Stranded mRNA and Total RNA Sample Preparation Kit (Illumina). Barcoded libraries were pooled at equal concentrations and sequenced on the HiSeq2000 or HiSeq2500 platform for 50 cycles. RNA-seq reads were trimmed of polyA tails, adapters and low-quality ends using Cutadapt^53^ with parameters --match-read-wildcards --times 2 -e 0 -O 5 --quality-cutoff' 6 -m 18 -b TCGTATGCCGTCTTCTGCTTG -b ATCTCGTATGCCGTCTTCTGCTTG -b CGACAGGTTCAGAGTTCTACAGTCCGACGATC -b TGGAATTCTCGGGTGCCAAGG -b AAAAAAAAAAAAAAAAAAAAAAAAAAAAAAAAAAAAAAAAAAAAAAAAAA -b TTTTTTTTTTTTTTTTTTTTTTTTTTTTTTTTTTTTTTTTTTTTTTTTTT . Reads were then mapped against a database of repetitive elements derived from RepBase (version 18.05) using Bowtie (version 1.0.0) with parameters -S -q -p 16 -e 100 -l 20 (ref. 54). Reads that did not map to Repbase sequences were aligned to the hg19 human genome (UCSC assembly) using STAR (version 2.3.0e)^55^ with parameters --outSAMunmapped Within –outFilterMultimapNmax 10 –outFilterMultimapScoreRange 1. Counts were calculated with featureCounts^56^ and reads per kilobase of transcript per million (RPKM) were computed. Differential expression was calculated using DESeq2 (ref. 57), individually pairing each knockdown experiments with their respective controls.

### Test of overlapping significance between gene sets

Genes from each differential expression experiment were considered significant if |log_2_ fold change| < log_2_(1.5) and the adjusted *P*<0.05. Significant genes between two sets were overlapped and the total set of genes was defined as genes that were expressed (RPKM>1) in the corresponding control experiment. A hypergeometic test was performed to determine whether the overlap of two gene sets was statistically significant. Regression analysis was performed using the scipy linear regression function on genes that were significantly differentially expressed in both samples.

### RT–PCR of splicing events

To validate AS events, RT–PCR (24–27 amplification cycles) was carried out using poly-A-selected and reverse-transcribed (Superscript III, Invitrogen) cDNA from mice (*n*=3) treated with either a control ASO or ASO targeting the indicated RBP. Isoform products were visualized using the Agilent 2200 TapeStation System (Agilent Technologies) or on an agarose gel and quantified using ImageJ to calculate ratios between inclusion and exclusion products. Statistical significance in differences between control and ASO samples was calculated by Student's *t*-test. Primer sequences are listed in Supplementary Data 9.

### qRT–PCR

qRT–PCR was performed using Power SYBR Green Master Mix (Life Technologies) using poly-A-selected and reverse-transcribed (Superscript III, Invitrogen) cDNA on an iQ5 real-time PCR detection system (Bio-Rad). For each biological replicate, qRT–PCR was carried out in technical triplicates. *GAPDH* and *Actin* were used as reference genes for human and mouse targets, respectively. Analysis was performed using the iQ5 optical system software (Bio-Rad; version 2.1). Expression values were normalized to the reference gene, and expression values were expressed as a fold change relative to control samples. Intergroup differences were assessed by two-tailed Student's *t*-test. Primer sequences were designed using the Primer3 software^58^ or obtained from PrimerBank^59^. Primer sequences are listed in Supplementary Data 9.

### RNA immunoprecipitation qPCR

NPCs were resuspended in lysis buffer (50 mM Tris pH 7.4, 100 mM NaCl, 1% NP-40, 0.1% SDS and 0.5% sodium deoxycholate) supplemented with 1 × Protease Inhibitor cocktail (Roche) and 80 U of RNAse Inhibitor (Roche). Clarified lysates were pre-cleared with Protein G agarose beads (Life Technologies). Aliquots of the supernatant (equivalent to 5% of supernatant) were saved as input protein and RNA. The remainder of the supernatant was incubated with 10 μg of antibody at 4 °C for 4 h. The protein–RNA–antibody complex was precipitated by incubation with Protein G magnetic beads overnight at 4 °C. Beads were washed twice with lysis buffer and three times with wash buffer (5 mM Tris pH 7.5, 150 mM NaCl, 0.1% Triton X-100). Ten per cent of the bead slurry was reserved for western blot analysis. The remaining bead slurry was resuspended in TRIzol (Life Technologies), and RNA was extracted as per the manufacturer's instructions. Input and immunoprecipitated RNA was converted into cDNA and gene expression was measured with qPCR. RNA immunoprecipitation qPCR studies were performed in biological duplicates. Primer sequences are listed in Supplementary Data 9.

### Antibodies for western blot analysis

The primary antibodies used are as follows: FUS/TLS (ProteinTech 1:1,000), FUS/TLS (Santa Cruz Biotechnology, clone 4H11, sc-47711, 1:100), TAF15 (Bethyl Laboratories 300A-308, 1:1,000), TDP-43 (Proteintech, 10782, 1:2,000) and GAPDH (Abcam, AB8245, 1:10,000). Images have been cropped for presentation. Full-size images are presented in Supplementary Fig. 8.

### Immunofluorescence

Cells were fixed in 4% paraformaldehyde for 20 min, washed three times in PBS and simultaneously blocked and permeabilized with 5% donkey serum and 0.1% Triton X-100 in PBS for 1 h at room temperature. Cells were then rinsed once in PBS and incubated with primary antibody overnight at 4 °C. After five washes with PBS, secondary antibodies consisting of goat anti-rabbit Alexa Fluor 488 and goat anti-mouse Alexa Fluor 555 (Life Technologies) were added at a dilution of 1:1,000 for 2 h at room temperature. Following incubation, the cells were rinsed three times with PBS, and nuclei were labelled with 1 μg ml^−1^ 4,6-diamidino-2-phenylindole for 10 min. The following primary antibodies were used: HB9 (1:100, DSHB), Islet1 (1:500, Santa Cruz Biotechnology), Oct4 (1:500, Cell Signaling), Olig2 (1:500, Millipore), Sox2 (1:500, Cell Signaling), Tra1-60 (1:1,000, Millipore), Tra1-81 (1:1,000, Millipore) and Tuj1 (1:500, Millipore).

### RBNS computational analysis

RBNS analysis was performed as previously described^60^. Briefly, motif enrichment (*R*) values were calculated for 6mers as the motif frequency in the RBP-selected pool over the frequency in the input RNA library. *R* values were considered significant if they had a *Z*-score ⩾ 2 (mean and s.d. calculated over all 6mers). Values in Fig. 2 and Supplementary Fig. 2 are for the protein concentration library with the highest overall enrichment (80 nM for both proteins). RBNS data sets have been deposited at the ENCODE DCC under accession IDs ENCSR936LOF for FUS and ENCSR827QYL for TAF15.

Motif logos were generated following an iterative procedure on the most enriched 6mer library precipitated from the GST-SBP-tagged protein: the most enriched 6mer was given a weight equal to its enrichment over the input library (=*R*−1), and all occurrences of that 6mer were masked in both the precipitated and input libraries. All enrichments were recalculated on the masked read sets to obtain the most enriched remaining 6mer and its corresponding weight, with this process continuing until the *R Z*-score was less than 2. All 6mers determined from this procedure were aligned to minimize mismatches to the most enriched 6mer, and a new motif was generated if the number of mismatches was greater than 2. The frequencies of each nucleotide in the position weight matrix, as well as the overall percentage of each motif, were determined from the weights of the individual aligned 6mers that went into that motif.

For comparison with CLIP-seq data, RBNS enrichments were determined from the concentration with the largest enrichment. For enrichment in CLIP-seq 6mers, FASTQ sequences were extracted from all clusters, and a matched number of random clusters from the same genomic region (5′ UTR, exon, 3′ UTR, proximal introns and distal introns). EMBOSS compseq was performed on the real and background set, and a delta between real and background *k*-mers was calculated with the equation:


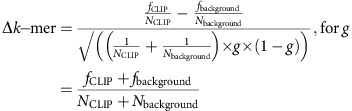


where *N* is the number of times the motif occurs in the set and *f* is observed frequency of the motif. To plot enrichment, all 6mers with the 4mer of interest were highlighted and a KDE plot was created for all 6mers. The Kolmogorov–Smirnov two-tailed test determined statistical significance in differences between distributions.

### RNA stability analysis

NPCs were infected with virus as described above. Ninety-six hours post infection, cells were treated with Actinomycin D (10 μg ml^−1^) for the indicated times. Cells were washed with cold PBS and harvested for RNA extraction using TRIzol (Life Technologies) or protein for western blot analysis. One microgram of total RNA was subjected to DNase treatment and poly(A) enrichment, and was used to prepare RNA-seq libraries as described above. To calculate RNA half-lives, RPKMs from each experiment were calculated and decay rates were generated by fitting RPKMs for each gene to a log-linear regression using the equation 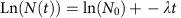 , where *t* is time and *N(t)* is the RPKM at time *t*. Half-lives were derived from the decay rate using the equation *t*_1/2_*=ln*(2)*/λ*. Genes were included in the analysis if their decay rate was positive (that is, RPKMs decreased over time) and the linear regression line had a *R*^2^ fit greater than 0.6.

### Correlation of gene expression to CLIP binding and motifs

Mouse brain CLIP-seq data for FUS and TDP-43 were previously described^21^,^22^. The binding location of each peak was assigned using the peak annotation method as described above. For each RBP, mouse brain CLIP-seq data and mouse striatum knockdown RNA-seq data were used to classify genes into the following categories: target and regulated, non-target and regulated, target and not regulated, and non-target and not regulated. A Fisher's exact test was performed to determine whether binding and regulation were significantly correlated. Motif analysis was performed similarly by determining whether a TAF15 ‘GGUAA' or FUS ‘GUGG' motif was present in the 3′ UTRs or introns of genes.

### Splicing-sensitive microarray analysis

Total RNA from three individual control and TAF15 ASO-treated mice were prepared for hybridization to splicing-sensitive microarrays (Affymetrix). Separation scores (Sep scores) were generated as previously described^61^. For clustering of splicing events, a splicing event was included in clustering if, for any of the three experiments, TAF15, FUS or TDP-43 knockdown was significantly (|Sep score|>0.5, *q* value<0.05) differentially expressed. Hierarchal clustering was performed using Seaborn/SciPy on the Sep scores for each splicing event. Overlap analysis of splicing-sensitive microarray results and TAF15 mouse CLIP-seq data was performed as previously described^61^.

### GO analysis

Significantly enriched GO terms were identified using a hypergeometric test that compared the number of genes that were either regulated (RNA-seq data) or bound (CLIP-seq data) in each GO term to genes expressed (background) in each GO term. The background gene set was defined as genes that were expressed (RPKM>1) in the corresponding control experiment.

### Data availability

The accession number for the sequencing data deposited in GEO for this paper is GSE77707.

## Additional information

**How to cite this article:** Kapeli, K. *et al*. Distinct and shared functions of ALS-associated proteins TDP-43, FUS and TAF15 revealed by multisystem analyses. *Nat. Commun.* 7:12143 doi: 10.1038/ncomms12143 (2016).

## Supplementary Material

Supplementary Information Supplementary Figures 1-8

Supplementary Data 1 Significantly differentially expressed genes upon TDP-43, FUS, or TAF15 knockdown in mouse striatum.

Supplementary Data 2 Significantly included and excluded splicing events in mouse striatum upon TDP-43, FUS, or TAF15 knockdown.

Supplementary Data 3 Stabilized and destabilized genes upon TAF15 or FUS knockdown in human neural progenitor cells.

Supplementary Data 4 Half-lives for genes upon TAF15, FUS, or control knockdown in human neural progenitor cells.

Supplementary Data 5 Gene ontology analysis of genes affected at the mRNA stability level upon TAF15 or FUS knockdown in neural precursor cells.

Supplementary Data 6 Significantly differentially expressed genes upon TDP-43, FUS, TAF15, or FUS+TAF15 knockdown in human iPSC-derived motor neurons.

Supplementary Data 7 Significantly differentially expressed genes in human patient FUS R521G iPSC-derived motor neurons vs. wild-type sibling control.

Supplementary Data 8 Significantly differentially expressed genes in sALS motor neurons vs. healthy control (non-ALS) motor neurons.
