# Supplementary material for: Distinct and shared functions of ALS-associated proteins TDP-43, FUS and TAF15 revealed by multisystem analyses
Source: Nat Commun. 2016 Jul 5;7:12143. doi: 10.1038/ncomms12143 (PMC4935974; doi:10.1038/ncomms12143)
Supplement: Supplementary Information — Supplementary Figures 1-8 [file ncomms12143-s1.pdf]

Supplemental Figures

Supplementary Figure 1

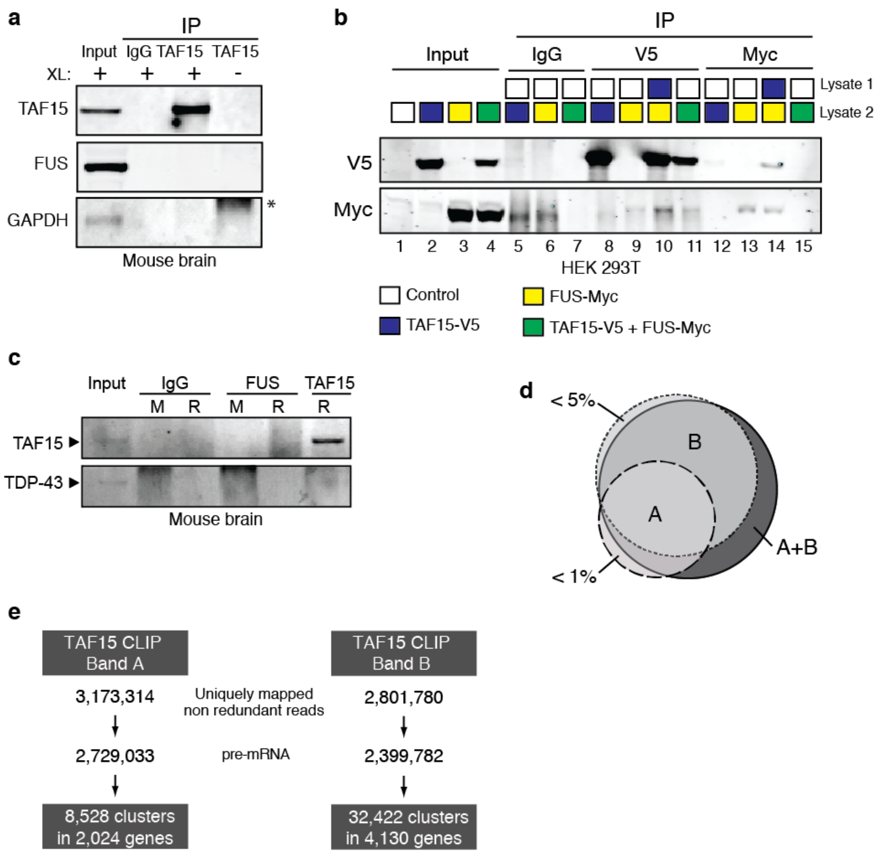

Supplementary Figure 1. CLIP-seq for TAF15 in the mouse brain. (a) Western blot to validate that FUS is not co-immunoprecipitated by TAF15 using an anti-TAF15 antibody from mouse brain tissue that was (+) or was not (-) subjected to UV crosslinking (XL). IgG antibody served as a negative control. The asterisk corresponds to a non-specific band. (b) Post-lysis interaction by ectopically expressed TAF15 and FUS. Cell lysates from HEK293T cells expressing an empty control plasmid (white), V5-tagged TAF15 (blue), Myc-tagged FUS (yellow), or both TAF15-V5 and FUS-Myc (green) were mixed in various paired combinations as indicated. Mixed lysates were used for immunoprecipitation with IgG, anti-V5, or anti-Myc antibodies and immunoprecipitated proteins were analyzed by Western blot for V5 and Myc expression. (c) Co-immunoprecipitation in mouse brain tissue that was subjected to UV crosslinking using two different anti-FUS, an anti-TAF15, or IgG control antibodies. Antibodies were either produced in mouse (M) or rabbit (R). (d) Venn diagram showing the overlap in CLIP peaks between the ‘A’ and ‘B’ TAF15-RNA complexes. (e) Flow-chart showing the breakdown of sequence reads for the ‘A’ and ‘B’ TAF15-RNA complexes.

Supplementary Figure 2

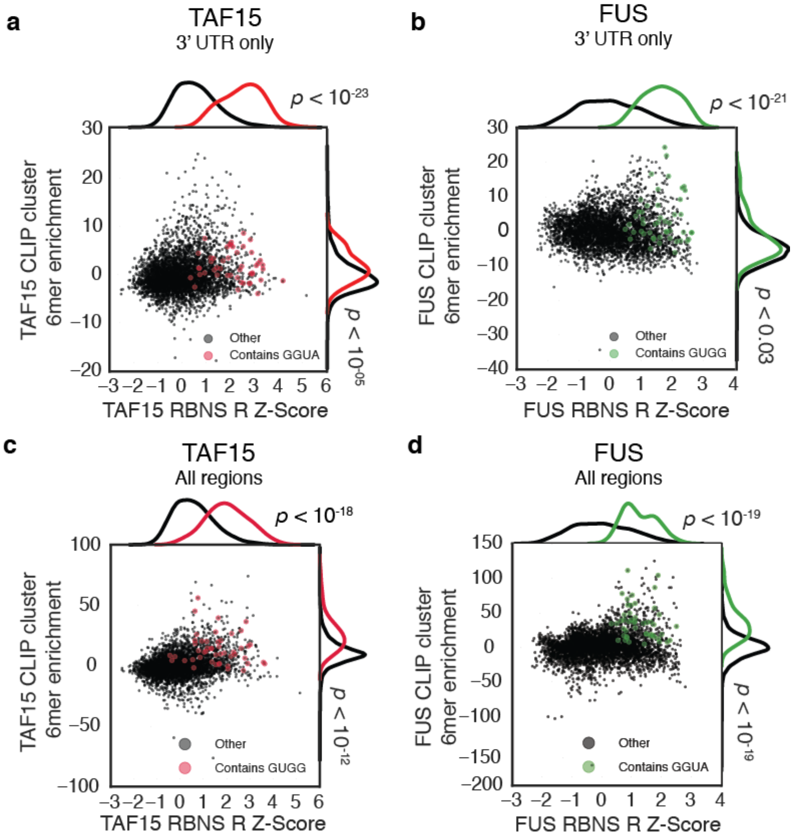

Supplementary Figure 2. RNA Bind-n-Seq confirms enrichment for GGUA motifs in RNAs that bind TAF15 *in vitro.* (a) Scatter plot correlating the percent enrichment above background of 6mers in TAF15 mouse brain CLIP-seq for peaks in the 3′UTR vs. TAF15 RBNS *R* Z-scores. Red dots represent all significant 6mers containing the GGUA motif. Histograms show normalized distributions of 6mers containing (red) or not containing (black) the GGUA motif in CLIP-seq (right) or RBNS (top). *p* values shown are computed by a Kolmogorov-Smirnov statistic. (b) Scatter plot as in (a) for 6mers in FUS mouse brain CLIP-seq peaks residing only in the 3′UTR vs. FUS RBNS *R* Z-scores. Green dots represent all significant 6mers containing the GUGG motif. (c) Scatter plot correlating the percent enrichment above background of 6mers in TAF15 mouse brain CLIP-seq vs. TAF15 RBNS *R* Z-scores. Red dots represent all significant 6mers containing the FUS GUGG motif. Histograms show normalized distributions of 6mers containing (red) or not containing (black) the GUGG motif in CLIP-seq (right) or RBNS (top). *p* values are shown. (d) Scatter plot as in (c) for 6mers in FUS mouse brain CLIP-seq vs. FUS RBNS *R* values. Green dots represent all significant 6mers containing the TAF15 GGUA motif.

Supplementary Figure 3

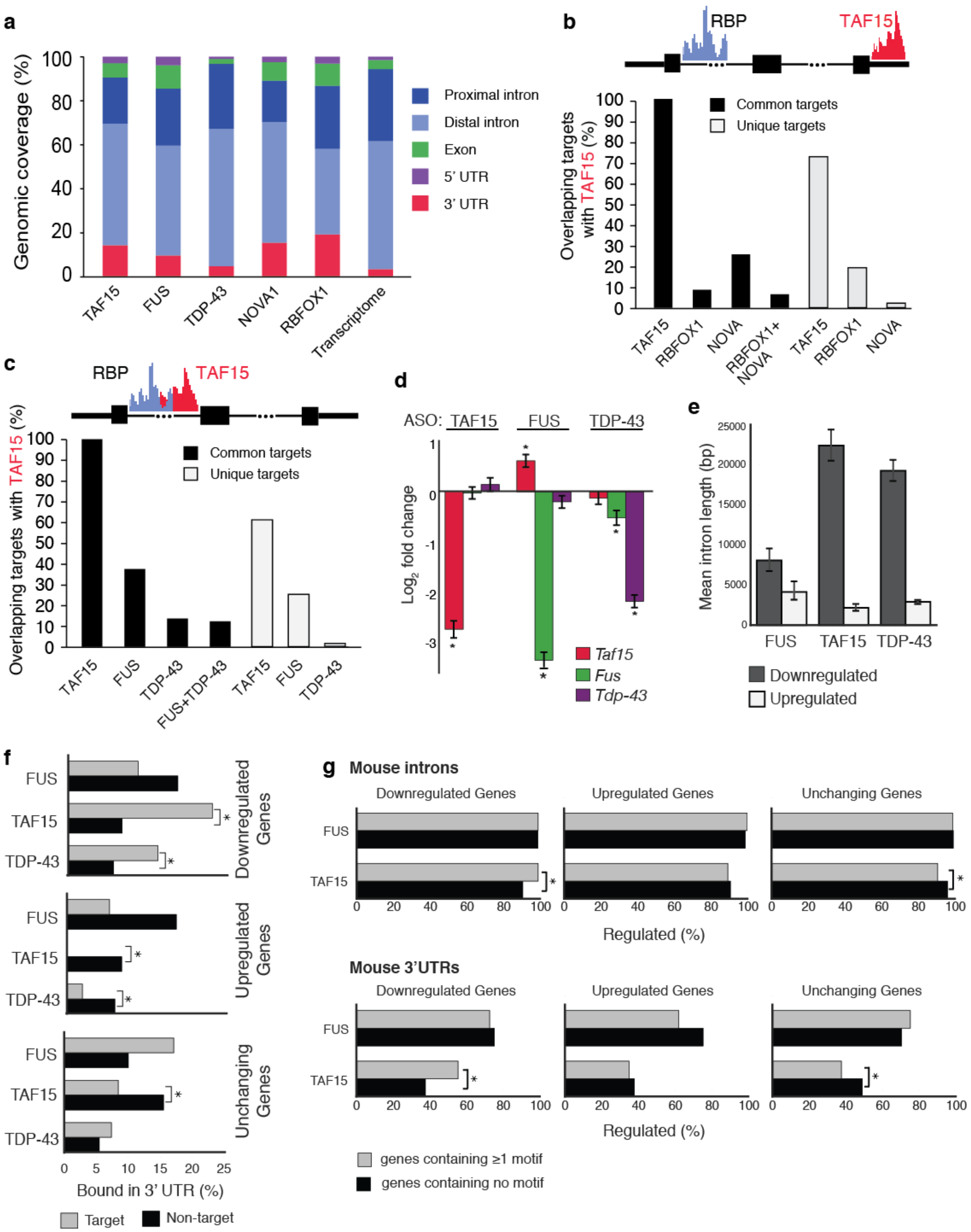

Supplementary Figure 3. TAF15 and FUS exhibit similar RNA interaction in the mouse brain. (a) Percent binding enrichment of TAF15, FUS, TDP-43, NOVA1 and RBFOX1 in proximal intron (dark blue), distal intron (light blue), exon (green), 5′UTR (purple) or 3′UTR (red) as compared to the observed percentage of nucleotides in the

annotated transcriptome. (b) Bar graphs showing the percent of gene targets that are common (black bars) or unique (gray bars) to TAF15 and NOVA1, RBFOX1, or both NOVA1 and RBFOX1. (c) Bar graph showing the percent of CLIP clusters that are common, i.e. within 100 bp of each other (black bars) or unique (gray bars) to TAF15 and FUS, TDP43, or both FUS and TDP-43. (d) Bar graph of log_2 fold change in gene expression (from RNA-seq, as measured by DESeq2) and the standard error for TAF15, FUS, and TDP-43 upon TAF15, FUS, or TDP-43 knockdown. (e) Mean intron length for all introns in genes that are either upregulated or downregulated by FUS, TAF15 or TDP-43. Error bars represent standard deviation. (f) Percent of genes that are downregulated, upregulated, or unchanged upon ASO-mediated knockdown of the indicated RBP that are either bound (Target, gray) or not bound (Non-target, black) by that RBP in the 3′UTR. Asterisks denote significant difference between target and non-target genes by Fisher's exact test. Error bars represent standard deviation.

Supplementary Figure 4

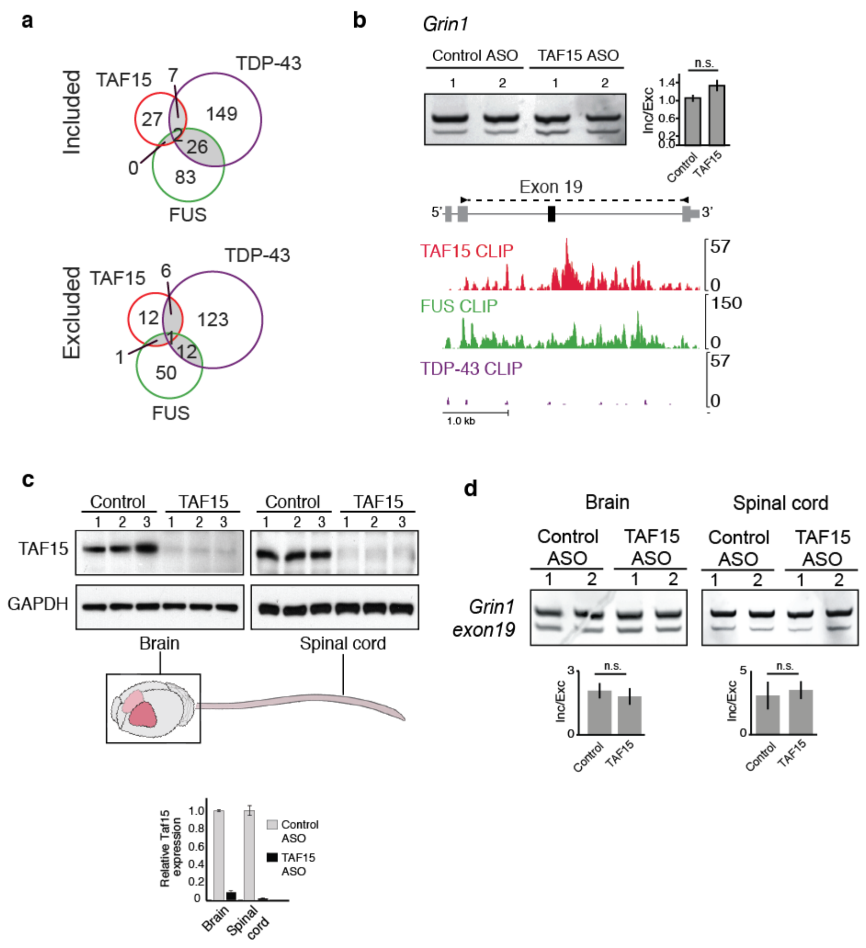

Supplementary Figure 4. Effect of TAF15 depletion on alternative splicing. (a) Venn diagrams showing common and unique inclusion (top) or exclusion (bottom) cassette events upon loss of FUS (green), TAF15 (red), or TDP-43 (purple). (b) RT-PCR of exon 19 of Glutamate receptor ionotropic, NMDA 1 (*Grin1;* chr2:25,294,438-25,294,549) to assess alternative splicing in TAF15 knockdown samples compared to controls. Quantification of biological replicates is shown. Error bars represent standard deviation. Binding of TAF15 (red), FUS (green), and TDP-43 (purple) in the mouse brain is shown below. (c) Confirmation of reduced TAF15 expression in the mouse brain or spinal cord by Western blot analysis (top) and qPCR (bottom). Knockdown was achieved by intracerebroventricular injection of ASOs complementary to TAF15 or a non-murine/human gene (Control). (d) Splicing validation by RT-PCR for exon 19 of *Grin1* upon ASO-mediated TAF15 depletion in the mouse brain or spinal cord compared to a Control ASO. Quantification of biological replicates is shown. Error bars represent standard deviation. n.s. indicates no significant difference.

Supplementary Figure 5

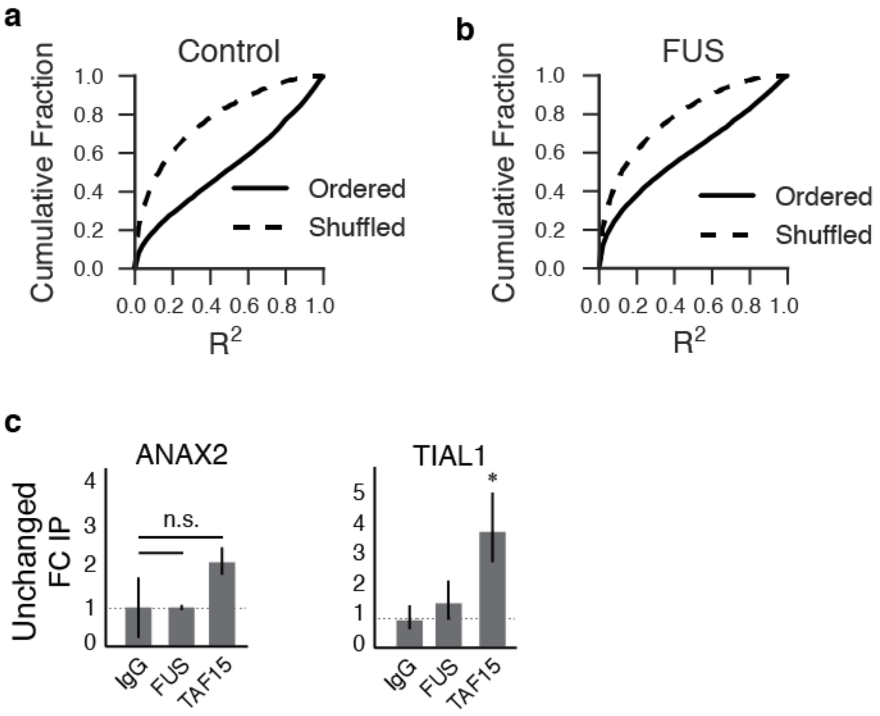

Supplementary Figure 5. Transcriptome-wide analysis of mRNA decay upon loss of TAF15 or FUS. (a, b) For each gene, the coefficient of determination (R^2) reflecting the fit of the RPKM values to a log linear regression was computed. The cumulative distribution functions of the R^2 values for all genes in the FUS depletion and scrambled shRNA treated experiments are depicted for real and shuffled values. (c) RNA immunoprecipitation was performed using antibodies against IgG (Control), TAF15 and FUS in NPCs. The relative fold change compared to the IgG control for *ANAX2* and *TIAL1*, RNAs that are not altered by TAF15 loss, was determined by qPCR. Values are means ± SD for biological duplicates. Asterisk denotes a significant difference compared to IgG by Student's *t* test where $p<0.05$ and n.s. indicates no significant difference.

Supplementary Figure 6

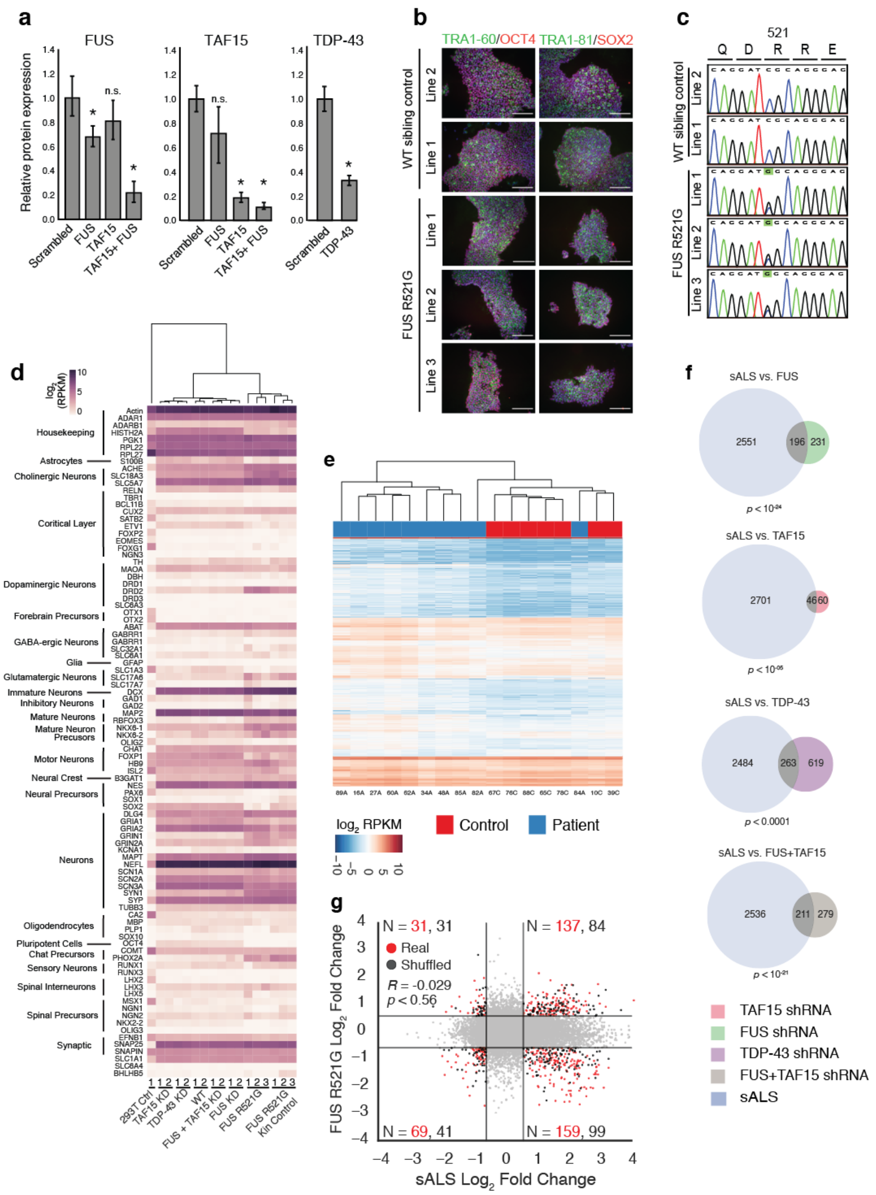

Supplementary Figure 6. Characterization of motor neuron model systems of sALS. (a). Densitometric quantification of Western blots in Fig. 6d. Asterisk denotes a significant difference compared to control (scramble) shRNA by Student's *t* test at $p<0.05$. Error bars represent standard deviation. (b) Immunofluorescence staining of ALS-patient and wild-type (WT) sibling control iPSCs for pluripotency markers TRA1-60, OCT4, TRA1-81, and SOX1. Dapi stain marks cell nuclei (blue). Scale bar = 100 μm. (c) Sanger sequencing results of iPSC lines in (b) showing the wild-type (c1561) or R521G mutation (c1561g) in the *FUS* gene. (d) Heatmap comparing the expression in $\log_2$ RPKMs of 93-gene RNA signature of glial, astrocyte, oligodendrocyte and neuronal

subtype markers in HEK293T cells, iPSC-derived human motor neurons (MNs) where TAF15, TDP-43, FUS, or both TAF15 and FUS have been knocked down (KD) in WT cells, and iPSC-derived MNs from ALS-patient fibroblasts harboring the FUS R521G mutation (FUS R521G) or the sibling control (FUS R521G Kin Control) and a scrambled shRNA treated FUS R521G Kin control (Line 3). Low expression is grey and high is purple. (e) Clustergram of log_2 RPKMs from sALS patient samples (blue) and control samples (red) used in the differential gene analysis. (f) Venn diagrams showing overlap of genes upregulated in sALS patient samples and genes downregulated upon loss of TAF15, FUS, TDP-43, or FUS+TAF15. *P* values were derived by hypergeometric test. (g) Scatter plot comparing gene expression changes in log_2RPKMs in MNs from sALS patient samples compared to expression in FUS R521G mutants. Each quadrant of a scatter plot shows genes (red dots) and gene counts (N, in red) that are significantly changing in sALS and RBP depletion experiments. Genes from a randomly ordered comparison are also shown (black dots) along with gene counts (N, in black). R^2 and *p* values from linear regression analyses of genes significantly changing in both sALS and RBP knockdown experiments are shown.

Supplementary Figure 7

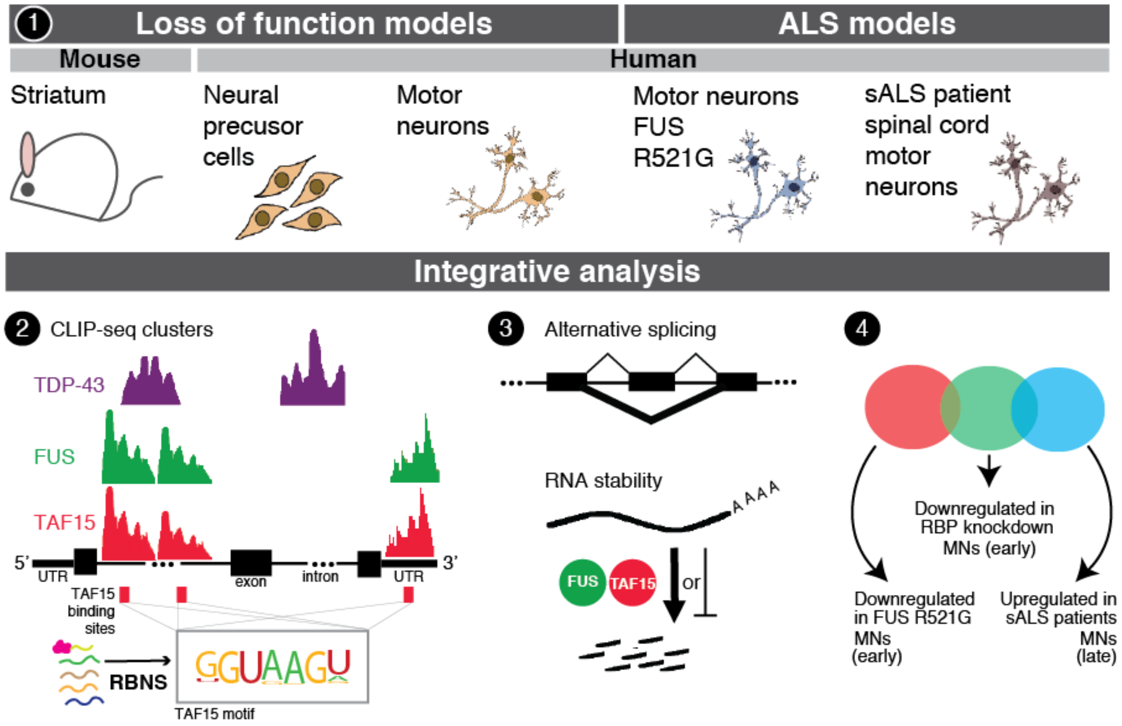

Supplementary Figure 7. Summary of findings. (1) Multiple model systems were employed to examine the neuronal functions of the ALS-associated RBPs TAF15, FUS, and TDP-43 through loss-of-function studies and compare these findings to models of ALS. Genome-wide studies such as (2) CLIP-seq revealed similar binding profiles for FUS and TAF15, but not TDP-43, and identified a novel TAF15 binding motif that was validated by RBNS. (3) TAF15 loss causes minimal changes in alternative splicing compared to FUS and TDP-43, but generally promotes exon skipping (bold line). TAF15 and FUS affect the stability of distinct mRNAs. (4) There is a small yet significant overlap of RNA signatures from motor neurons (MNs) depleted of TAF15, FUS, or TDP-43 with MNs differentiated from FUS R521G ALS patient fibroblasts (representative of an early stage of sALS) or obtained from sALS patient spinal cords (representative of a late stage of sALS).

Supplementary Figure 8

Figure 3. Western blot

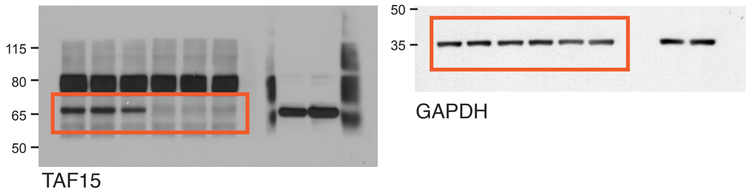

Figure 4. TapeStation image of *Gpcpd* (c) and *Kcnma1* (d).

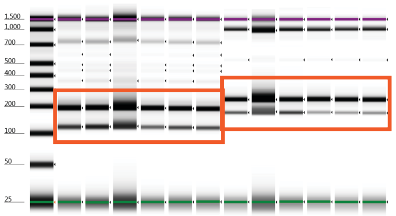

Figure 5. Western blot images

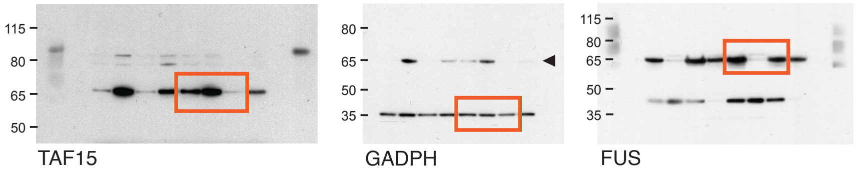

► TAF15 signal from previous antibody probing.

Figure 6. Western blot images.

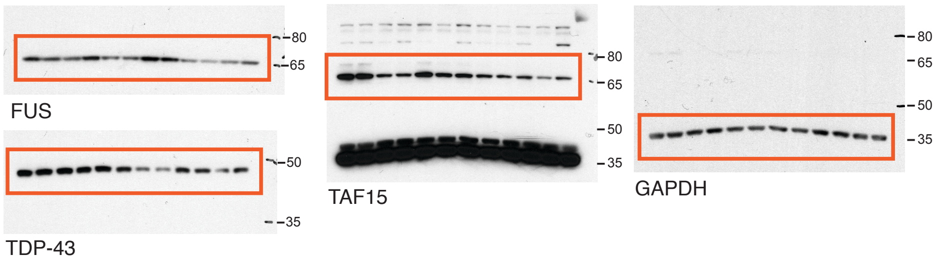

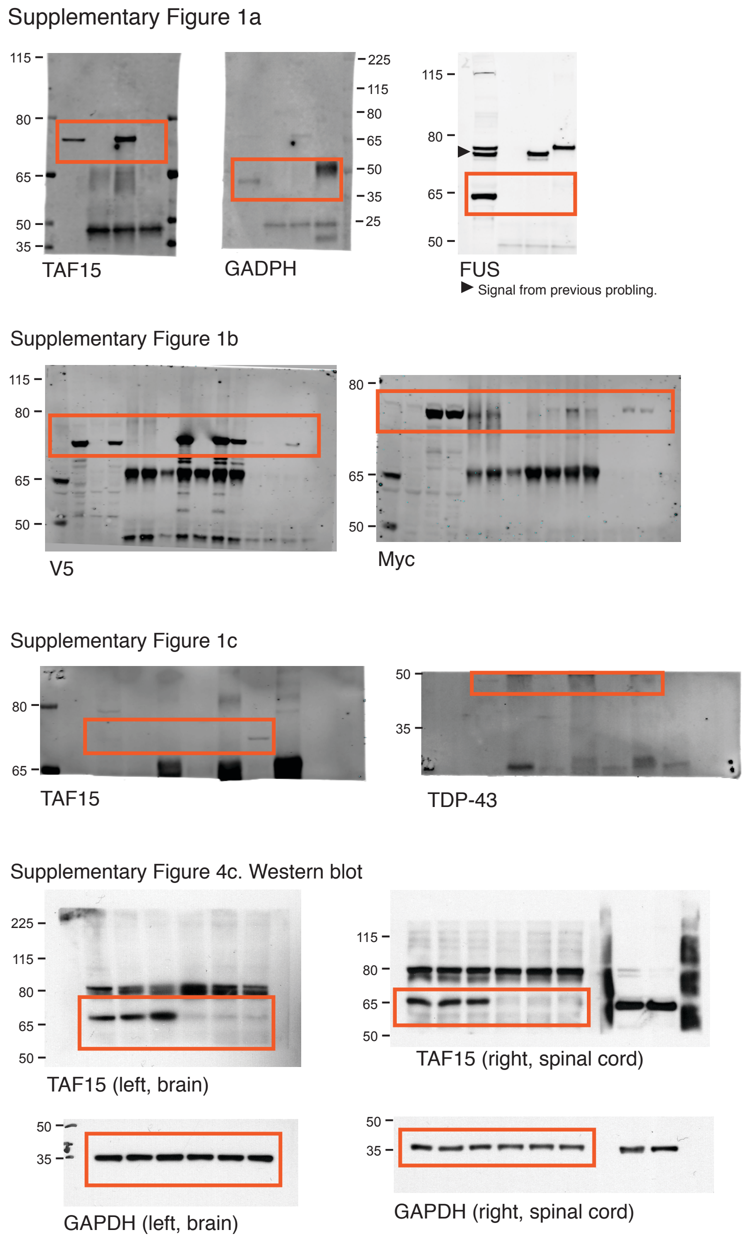

Supplementary Figure 8. Images of uncropped figures. Orange boxes indicate cropped images shown in main and supplemental figures.
